# Calsequestrin 2 overexpression in breast cancer increases tumorigenesis and metastasis by modulating the tumor microenvironment

**DOI:** 10.1002/1878-0261.13136

**Published:** 2021-11-14

**Authors:** Ju Hee Kim, Eun‐Shin Lee, Jihui Yun, Han Suk Ryu, Hong Kyu Kim, Young Wook Ju, Kwangsoo Kim, Jong‐Il Kim, Hyeong‐Gon Moon

**Affiliations:** ^1^ Biomedical Research Institute Seoul National University Hospital South Korea; ^2^ Department of Pathology Seoul National University School of Medicine South Korea; ^3^ Genomic Medicine Institute Medical Research Center Seoul National University Korea; ^4^ Department of Biomedical Sciences Seoul National University College of Medicine Korea; ^5^ Department of Pathology Seoul National University Hospital South Korea; ^6^ Department of Surgery Seoul National University Hospital Korea; ^7^ Division of Clinical Bioinformatics Seoul National University Hospital Korea; ^8^ Cancer Research Institute Seoul National University Korea; ^9^ Department of Biochemistry and Molecular Biology Seoul National University College of Medicine Korea; ^10^ Department of Surgery Seoul National University College of Medicine South Korea

**Keywords:** breast cancer, calsequestrin 2, metastasis, tumor microenvironment

## Abstract

The spatial tumor shape is determined by the complex interactions between tumor cells and their microenvironment. Here, we investigated the role of a newly identified breast cancer‐related gene, calsequestrin 2 (*CASQ2*), in tumor–microenvironment interactions during tumor growth and metastasis. We analyzed gene expression and three‐dimensional tumor shape data from the breast cancer dataset of The Cancer Genome Atlas (TCGA) and identified CASQ2 as a potential regulator of tumor–microenvironment interaction. In TCGA breast cancer cases containing information of three‐dimensional tumor shapes, *CASQ2* mRNA showed the highest correlation with the spatial tumor shapes. Furthermore, we investigated the expression pattern of *CASQ2* in human breast cancer tissues. CASQ2 was not detected in breast cancer cell lines *in vitro* but was induced in the xenograft tumors and human breast cancer tissues. To evaluate the role of CASQ2, we established *CASQ2*‐overexpressing breast cancer cell lines for *in vitro* and *in vivo* experiments. *CASQ2* overexpression in breast cancer cells resulted in a more aggressive phenotype and altered epithelial–mesenchymal transition (EMT) markers *in vitro*. *CASQ2* overexpression induced cancer‐associated fibroblast characteristics along with increased hypoxia‐inducible factor 1α (HIF1α) expression in stromal fibroblasts. *CASQ2* overexpression accelerated tumorigenesis, induced collagen structure remodeling, and increased distant metastasis *in vivo*. CASQ2 conferred more metaplastic features to triple‐negative breast cancer cells. Our data suggest that CASQ2 is a key regulator of breast cancer tumorigenesis and metastasis by modulating diverse aspects of tumor–microenvironment interactions.

## Graphical abstract

CASQ2 is a potential regulator of the tumor–microenvironment interaction. CASQ2 was identified through correlation analysis of tumor three‐dimensional shapes. Overexpression of *CASQ2* in breast cancer cells resulted in more aggressive phenotypes and dysregulation of intracellular signaling pathways, along with increased tumorigenesis and remodeling of collagen structures. *In vivo*, *CASQ2* was linked to HIF‐1 signaling and increased metastatic colonization. Thus, CASQ2 conclusively conferred breast cancer cells with more metaplastic features.

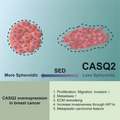

**Abbreviations**
CASQ2: calsequestrin 2
CPVT: catecholaminergic polymorphic ventricular tachycardia
ECM: extracellular matrix
EMT: epithelial–mesenchymal transition
HIF‐1: hypoxia‐inducible factor
MBC: metaplastic breast carcinoma
SED: spheroid–ellipsoid discrepancy
SHG: second‐harmonic generation
TACS: tumor‐associated collagen signature
TCGA: The Cancer Genome Atlas
TME: tumor microenvironment

## 1 Introduction

Breast cancer is the most commonly diagnosed cancer and the leading cause of cancer‐related deaths in over 100 countries [1]. Metastasis is a major hallmark and the main determinant of lethality associated with breast cancer [2]. The spatial growth of solid tumor cells is known to reflect their ability to invade and migrate during metastasis. Despite their common origin, tumors might have unique characteristics related to their spatial heterogeneity. This intratumoral morphological heterogeneity of different subclonal tumor populations might be related to genetic, phenotypic, or behavioral characteristics [3, 4, 5]. The spatial growth of tumors depends on several cell types and is a result of continuous interactions between tumors and the tumor microenvironment (TME) [6, 7].

Calsequestrin 2 (CASQ2) binds to calcium and is located in the intracellular endoplasmic reticulum or sarcoplasmic reticulum in cardiomyocytes and skeletal muscle [8]. Particularly, CASQ2 has been shown to modulate muscle contraction and activate cardiomyocytes by regulating the intracellular concentration of calcium. Moreover, CASQ2 is the major calcium reservoir protein in the heart, sensing the concentration of calcium and releasing it to the cytosol through ryanodine receptor 2 (RyR2) [9].

As tumors are surrounded by the extracellular matrix (ECM) and stromal cells, the physiological state of the TME has an effect on all stages of tumorigenesis [10]. The TME comprises different cell types, and the tumor mass might thus be determined by a gradient of oxygen and nutrient levels under the influence of growth factors and cytokines. Calcium signaling is involved in various important processes related to tumor progression, such as proliferation, migration, and invasion [11, 12]. The altered expression of calcium‐regulating proteins has been reported in breast cancer cell lines [13]. The calcium channel protein, TRPV4, has been reported to mediate the calcium ion (Ca^2+^) influx during the migration of endothelial cells in breast cancer [14]. In addition, intracellular Ca^2+^ oscillation is significantly enhanced in osteoclast (bone resorption) progenitor cells after stimulation with media conditioned by tumorigenic breast cancer cells, demonstrating the importance of calcium signaling in TME stromal cells [15].

Metaplastic breast carcinoma is a rare neoplasm and a distinct aggressive and invasive form of triple‐negative breast cancer, with histological evidence of epithelial‐to‐mesenchymal transition (EMT) toward spindle, chondroid, or osseous cell types [16]. Proteome analysis of human spindle metaplastic breast carcinoma has revealed genetic alterations in calcium binding and ECM organization [17].

As tumor–stromal interactions at the tumor invasive front are major determinants of spatial tumor growth [18], we reasoned that unraveling the gene expression features associated with the spatial tumor shape could help identify genes critical in the tumor–stromal interaction. Thus, we reviewed the pathology reports of breast tumors on The Cancer Genome Atlas (TCGA) to determine the association of the expression levels of genes with tumor shape. Based on these results, we further explored the role of candidate genes in determining tumor shape using *in vitro* and *in vivo* models.

## 2 Materials and methods

### 2.1 Human tissue samples

The acquisition of human tissues was approved by the Institutional Review Board of Seoul National University Hospital (Seoul, South Korea, SNUH IRB number: 1712‐141‐909). All procedures were performed in accordance with the Declaration of Helsinki. Written informed consent was obtained from all patients. Patients were included in this study if they met the following criteria: patients with invasive breast cancer with three‐dimensional tumor shape information and patients who did not receive neoadjuvant therapies. The following is the number of samples used for data analysis: CASQ2 RNA‐sequencing data of SNUH patients, 32; CASQ2 mRNA levels in SNUH patients from quantitative real‐time PCR, 143; and CASQ2 IHC staining, 5.

### 2.2 Correlation between the expression of tumor genes and roundness

TCGA breast cancer gene expression data were downloaded from TCGA FireBrowse [19]. RSEM values were selected as gene expression values. Spearman's correlation was then used to evaluate the correlation between the gene expression values in breast cancer samples and the corresponding spheroid–ellipsoid discrepancy (SED) values. Only genes with a significant correlation and *P < *0.05 after Benjamini–Hochberg multiple testing correction were considered for further analysis [20].

### 2.3 TCGA and METABRIC data analysis

TCGA *z*‐scores were classified as low and high expressions of genes with cutoff values of < 0.0 and ≥ 0.0, respectively. Tumor SED values were classified as more spheroid and less spheroid with cutoff values of < 0.5 and ≥ 0.5, respectively. Gene set enrichment analysis (GSEA) of TCGA was performed using Cancer Gene and Pathway Explorer (CGPE) [21]. METABRIC breast cancer expression data were downloaded from cBioPortal (http://www.cbioportal.org/). METABRIC data were also analyzed according to the same cutoff used for TCGA data analysis.

### 2.4 Cell culture, migration, and invasion assays

Breast cancer cell lines and WI‐38 cells were obtained from the American Type Culture Collection (ATCC, VA, USA). Cells were cultured in Dulbecco's modified Eagle’s medium (Welgene, Seoul, South Korea) containing 10% fetal bovine serum (Welgene) and 1% penicillin/streptomycin (Gibco, Waltham, MA, USA). For migration assays, 2 × 10^5^ cells were seeded in an insert (8‐μm pore size, Corning Incorporated, Corning, NY, USA) in a serum‐free medium. For invasion assays, 1 mg·mL^−1^ Matrigel was added to an insert before seeding cells. Medium supplemented with 10% fetal bovine serum was added to the lower chambers. Cells were incubated for 24 h, fixed with 4% paraformaldehyde (Biosesang, Seoul, South Korea), and stained with 0.1% crystal violet (Sigma‐Aldrich, St. Louis, MO, USA). Quantitative evaluation of migrated and invaded cells was performed using imagej (Java 1.8.0_172) software (NIH, Bethesda, MD, USA).

### 2.5 Proliferation assay

Cells were plated on flat‐bottom 96‐well culture plates (1 × 10^4^ cells per well) and incubated for 4 h with 0.5 mg·mL^−1^ thiazolyl blue tetrazolium bromide (Sigma‐Aldrich). The resulting methylthiazole tetrazolium formazan crystals were treated with dimethyl sulfoxide (Duchefa Biochemie, Haarlem, The Netherlands). The absorbance of the sample at 540 nm was measured using a microplate reader (BioTek Instruments, Winooski, VT, USA).

### 2.6 Three‐dimensional culture and mammosphere culture assay

For three‐dimensional cell cultures, the cells were seeded on growth factor‐reduced Matrigel (BD Biosciences, San Jose, CA, USA). For mammosphere culture, the cells were suspended at 10 000 cells·mL^−1^ and seeded in six‐well ultra‐low attachment plates (Corning Incorporated) in MammoCult medium (STEMCELL Technologies, Vancouver, BC, Canada).

### 2.7 Measurement of intracellular levels of calcium

The cells (1 × 10^5^/well) were seeded on flat‐bottom black 96‐well culture plates. After 24 h, they were incubated in serum‐free Dulbecco’s modified Eagle’s medium for 4 h, washed twice with regular buffer [10 mm HEPES (pH 7.4), 140 mm NaCl, 10 mm glucose, 5 mm KCl, 1 mm CaCl_2_, and 1 mm MgCl_2_], and treated with 5 μm calcium crimson in regular buffer for 30 min at 25 °C. For calcium chelation, the cells were incubated with 10 μm BAPTA‐AM (Thermo Fisher Scientific, Waltham, MA, USA) for 15 min in regular buffer at 37 °C and 5% CO_2_, and then, the BAPTA‐AM loading solution was removed and the cells were treated with calcium crimson as described above. After buffer replacement with calcium‐free buffer [10 mm HEPES (pH 7.4), 140 mm NaCl, 10 mm glucose, 5 mm KCl, 1 mm CaCl_2_, 1 mm MgCl_2_, 1 mm EDTA, and 1 mm EGTA], the cells were stimulated with 5 µm caffeine. The level of intracellular calcium was determined with the 590/615‐nm ratio (emission at 590‐nm excitation/emission at 615‐nm excitation) using a multimode microplate reader (BioTek Instruments).

### 2.8 Quantitative RT‐PCR

The total RNA was extracted from the cells lysed by TRIzol (Favorgen, Pingtung, Taiwan). The Prime Script 1st strand cDNA Synthesis Kit (Takara Bio, Kusatsu, Japan) was used for the reverse transcription of RNA, and then, cDNA was amplified using Power SYBR^®^ Green PCR Master Mix (Applied Biosystems, Waltham, MA, USA). The following primer sequences (5′‐3′) were used: *CASQ2*, (forward) GGAACACCAAAGACCCACTC, (reverse) TTCTCTGCAAAGGCCACAAT; *αSMA*, (forward) CAAAG CCGGCCTTACAGAG, (reverse) AGCCCAGCCAAGCACTG; *FSP1*, (forward) AACTTGTCACCCTCT TTGCC, (reverse) TCCTCAGCGCTTCTTCTTTC; *VIMENTIN*, (forward) GCAAAGATTCCACTTTGCGT, (reverse) GAAATTGCAGGAGGAGATGC; *HIF1α*, (forward) AATTCTCAACCACAGTGCATTGTATGT, (reverse) CTTTGGTGAATACCTGACTCATTTTCA; *COL2A1*, (forward) GCAGGATGGGCA GAGGTAT AA, (reverse) CGAGGTCAGTTGGGCAGATG; *CPL4A1*, (forward) GCACAGCCAGACCATTCAGAT, (reverse) GCGCACTTCTAAACTCCTCCA; *SOX9*, (forward) CAGCGAACGCACATCAAGAC, (reverse) AGTTCTGGTGGTCGGTGTAGT; *COL1A1*, (forward) AAGAACAGCGTGGCCTACATG, (reverse) GG GAGGTCTTGGTGGTTTTGT; *SP7*, (forward) TGCTTGAGGAGGAAGTTCAC, (reverse) AGGTCACTGCCCACAGAGTA; *FN1*, (forward) CAAGCCAGATGTCAGAAGC, (reverse) GGATGGTGCATCAATG GCA; and *GAPDH*, (forward) GAGTCCACTGGCGTCTTC, (reverse) GGAGGCATTGCTGATGATC.

### 2.9 Immunoblotting

The cells (3 ×fnd="ER"> 10^5^/60‐mm culture dish) were lysed in RIPA buffer containing a protease and phosphatase inhibitor cocktail (Thermo Fisher Scientific). The proteins in the cell lysates, separated by sodium dodecyl sulfate/polyacrylamide gel electrophoresis, were transferred onto polyvinylidene fluoride membranes (MERCK, St. Louis, MO, USA). After membrane incubation with specific antibodies, the signal was enhanced with chemiluminescence reagents (Thermo Fisher Scientific) and then measured using an Amersham Imager 680 (GE Healthcare Life Sciences, Pittsburgh, PA, USA). The following primary antibodies were used: anti‐CASQ2 (#sc‐390999; Santa Cruz Biotechnology, Dallas, TX, USA), anti‐HIF1α (#NB100‐131; NOVUS, Littleton, CO, USA), anti‐vimentin (#5741; Cell Signaling Technology, Danvers, MA, USA), anti‐pERK (#9102; Cell Signaling Technology), anti‐ERK (#9106; Cell Signaling Technology), anti‐pAKT (#9271; Cell Signaling Technology), anti‐AKT (#9272; Cell Signaling Technology), anti‐pmTOR (#2971; Cell Signaling Technology), anti‐mTOR (#2983; Cell Signaling Technology), anti‐slug (#9585; Cell Signaling Technology), anti‐snail (#3879; Cell Signaling Technology), anti‐N‐cadherin (#13116; Cell Signaling Technology), anti‐ZEB (#33969; Cell Signaling Technology), anti‐aSMA (#BS70000; Bioworld Technology, St. Louis Park, MN, USA), anti‐FSP1 (#BS7671; Bioworld Technology), anti‐CD24 (#AF5247; R&D Systems, Minneapolis, MN, USA), anti‐CD44 (#51037; Abcam, Cambridge, UK), and anti‐ALDH (#611194; BD Biosciences).

### 2.10 Establishment of stable breast cancer cell lines using retroviruses

To prepare human and mouse *CASQ2*‐overexpressing cell lines, retroviruses encoding human *CASQ2* gene were produced using the pMSCV‐GFP or pMSCF‐RFP vector (Clontech, Kusatsu, Japan). HEK293FT cells were transfected with pMSCV‐GFP‐hCASQ2 or pMSCV‐RFP‐hCASQ2, pgag‐pol, and VSV‐G, using Lipofectamine 3000 (Thermo Fisher Scientific). After 48 h, the medium containing the retroviruses was collected and filtered to remove cell debris. The breast cancer cell lines were infected following inoculation with the retroviruses, and cells expressing CASQ2 were selected using puromycin (Thermo Fisher Scientific).

### 2.11 Indirect coculture

Cancer cells (Hs578T, 2 × 10^5^/well) were seeded in six‐well plates ensuring that after 48 h, the cells were not overconfluent. Transwells (pore size 0.4 μm, Corning) were placed on top of the cancer cells, and fibroblasts (WI‐38, 2 × 10^5^/well) were plated in each Transwell at the same cell concentration. In the opposite case, the position of the cells in the six‐well plates and Transwell was reversed. The cells were harvested after incubation for 48 or 72 h.

### 2.12 Immunohistochemistry assay

Immunohistochemistry was performed using the IHC Staining Kit (Dako, Santa Clara, CA, USA). Tissue sections were deparaffinized in xylene (Thermo), hydrated in phosphate‐buffered saline (Welgene), and blocked with Background Reducing Solution (Dako). The sections were incubated with anti‐CASQ2 (#NBP1‐87304; NOVUS), anti‐aSMA (#BS70000; Bioworld Technology), anti‐FSP1 (#BS7671; Bioworld Technology), anti‐HIF1α (#NB100‐131; NOVUS), anti‐vimentin (#5741; Cell Signaling Technology), anti‐pan‐cytokeratin (#M3515; Dako), and anti‐ki67 (#9027; Cell Signal Technology) at 4 °C overnight, followed by incubation with horseradish peroxidase‐conjugated anti‐secondary antibody (Dako). The signal was developed using diaminobenzidine and hydrogen peroxide, resulting in a brown precipitate. The sections were counterstained with hematoxylin (Dako), dehydrated, and mounted. The DAB area was quantified using IHC Toolbox in ImageJ software (NIH) with 20 random histological fields from five slides of tumor tissues per group [22].

### 2.13 Immunofluorescence and microscopy

For immunofluorescence and confocal microscopy, the cells (0.5 × 10^5^/well) were grown on two‐well chamber slides, rinsed with PBS, and fixed for 20 min with 4% paraformaldehyde at room temperature. After rinsing with PBS, the cells were permeabilized with 0.1% Triton X‐100 in PBS for 5 min and rinsed with PBS. F‐Actin stained with Alexa 488/phalloidin (Invitrogen, Waltham, MA, USA) for 1 h at room temperature, and then with DAPI. The cells were then washed extensively with PBS, rinsed briefly with distilled water, and mounted on a glass slide in mounting reagent (Vector Laboratories, Burlingame, CA, USA). The cells were viewed with a confocal microscope (Leica TCS SP8; Leica, Wetzlar, Germany) at Ex/Em 493/517 nm.

### 2.14 Imaging of collagen fibers and analysis

A laser‐scanning two‐photon microscope (IVM‐M; IVIM Technology, Daejeon, Korea) was used to visualize collagen fibers in *ex vivo* tumor samples. The collagen fibers were imaged by intrinsic second‐harmonic generation (SHG) signals generated from the collagen fibers with a highly noncentrosymmetric structure. Hs578T tumors were imaged by fluorescence signals of transfected red fluorescence protein (RFP). The SHG and fluorescence signals were excited by Ti:sapphire femtosecond pulse laser (Chameleon Vision S; Coherent, Santa Clara, CA, USA) tuned at 840 nm, and simultaneously detected using photomultiplier tubes (PMTs) equipped with bandpass filters (415–425 nm for SHG and 565–605 nm for RFP). For volumetric imaging, z‐stack images (FOV; 500 μm × 500 μm) with 5‐μm z‐axial spacing were acquired from the tumor samples using a commercial objective lens (CFI Plan Apo Lambda 20×; Nikon, Minato‐ku, Tokyo, Japan). Collagen deposition in each group was quantified by measuring the average SHG signal intensity or collagen‐deposited area ratio at the central area of 10 sequential z‐stack images. Average SHG signal intensity was calculated using imagej measurement plugins (NIH). Collagen‐deposited area ratio was calculated by identifying the SHG signal‐positive area over the total imaging area. The other collagen parameters (angle and straightness) were assayed using CT‐FIRE, an open‐source software package specifically designed for automatic quantification of collagen fibers within SHG images (http://loci.wisc.edu/software/ctfire).

### 2.15 RNA sequencing

The total RNA was obtained from control or *CASQ2*‐overexpressing Hs578T cells (3 × 10^5^/ 60‐mm culture dish, Hs578T‐CTL and Hs578T‐CASQ2‐o/e) or xenograft tumor tissues. An RNA sequence library was prepared for each cDNA sample using the Illumina TruSeq‐Stranded mRNA Sample Prep Kit (Illumina, San Diego, CA, USA) according to the manufacturer’s protocol. The FPKM transcript was used as a reference for transcript expression. Differentially expressed genes (DEGs) with adjusted *P < *0.05, log2 fold‐change ≥ 1, and average of expression (FPKM) across all samples ≥ 1 were selected (*n* = 530). To identify significantly enriched pathways (Kyoto Encyclopedia of Genes and Genomes, KEGG) from DEGs, we used DAVID Bioinformatics Database Functional Annotation Tool (http://david.abcc.ncifcrf.gov/). Results with *P < *0.05 were considered statistically significant.

### 2.16 Chondrogenesis and osteogenesis assay

The cells (2 × 10^5^ cells) were grown in chondrogenic or osteogenic differentiation medium (R&D Systems) for 14 days according to the manufacturer’s protocol, and then, the pellets were harvested for RNA precipitation. Chondrogenic pellets were frozen‐sectioned, and analyzed using an Alcian blue staining kit (Vector Laboratories). The stained regions were calculated using imagej (Java 1.8.0_172) software (NIH).

### 2.17 Xenograft/allograft tumor mouse model

Five‐week‐old female mice were used for *in vivo* experiments. All procedures were approved by the Association for Assessment and Accreditation of Laboratory Animal Care. The animal care and use protocol was reviewed and approved by the Institutional Animal Care and Use Committee of Seoul National University Hospital (no. 19‐0209‐S1AO), and animals were maintained in the facility‐accredited AAALAC International (#001169) in accordance with Guide for the Care and Use of Laboratory Animals 8th edition, NRC (2010). 4T1 cells (1 × 10^5^ cells) were injected into the mammary fat pads of BALB/c (Orient, Seoul, South Korea). MDA‐MB‐231 cells (1 × 10^6^ cells) were injected into mammary fat pads of immune‐deficient BALB/c nude mice (Orient). Hs578T‐CTL, Hs578T‐CASQ2 o/e, MDA‐MB‐231‐CTL, and MDA‐MB‐231‐CASQ2 o/e (1 × 10^6^ cells) were injected into the mammary fat pads of NOD/SCID mice (NSG, Orient). For experimental lung metastasis, the cells were intravenously injected into the tail vein. Tumor size was measured using a caliper, and tumor volume was calculated using the following formula: 0.523 × length × width^2^ (mm^3^). Metastatic areas were calculated using imagej (Java 1.8.0_172) software (NIH).

### 2.18 Statistical analysis

Statistical analyses were performed using graphpad prism 8 (GraphPad Software, San Diego, CA, USA). Data are expressed as mean ± standard error of the mean (SE). Analysis of variance, ANOVA with Turkey’s *post hoc* test, and the multiple *t*‐test were used to compare the mean among three or more groups, as well as tumor volume. Student’s *t*‐test or the Mann–Whitney *U*‐test was performed to compare the mean between two groups as appropriate. The log‐rank test for trend was used to compare Kaplan–Meier curves. The chi‐square test was used to assess relationships among factorial variables. The following significance levels were used: ns, not significant, *P < 0.05; **P < 0.01; ***P < 0.001; and ****P < 0.0001.

## 3 Results

### 3.1 Identification of CASQ2 as a potential regulator of spatial tumor shape in breast cancer

**Fig. 1:**
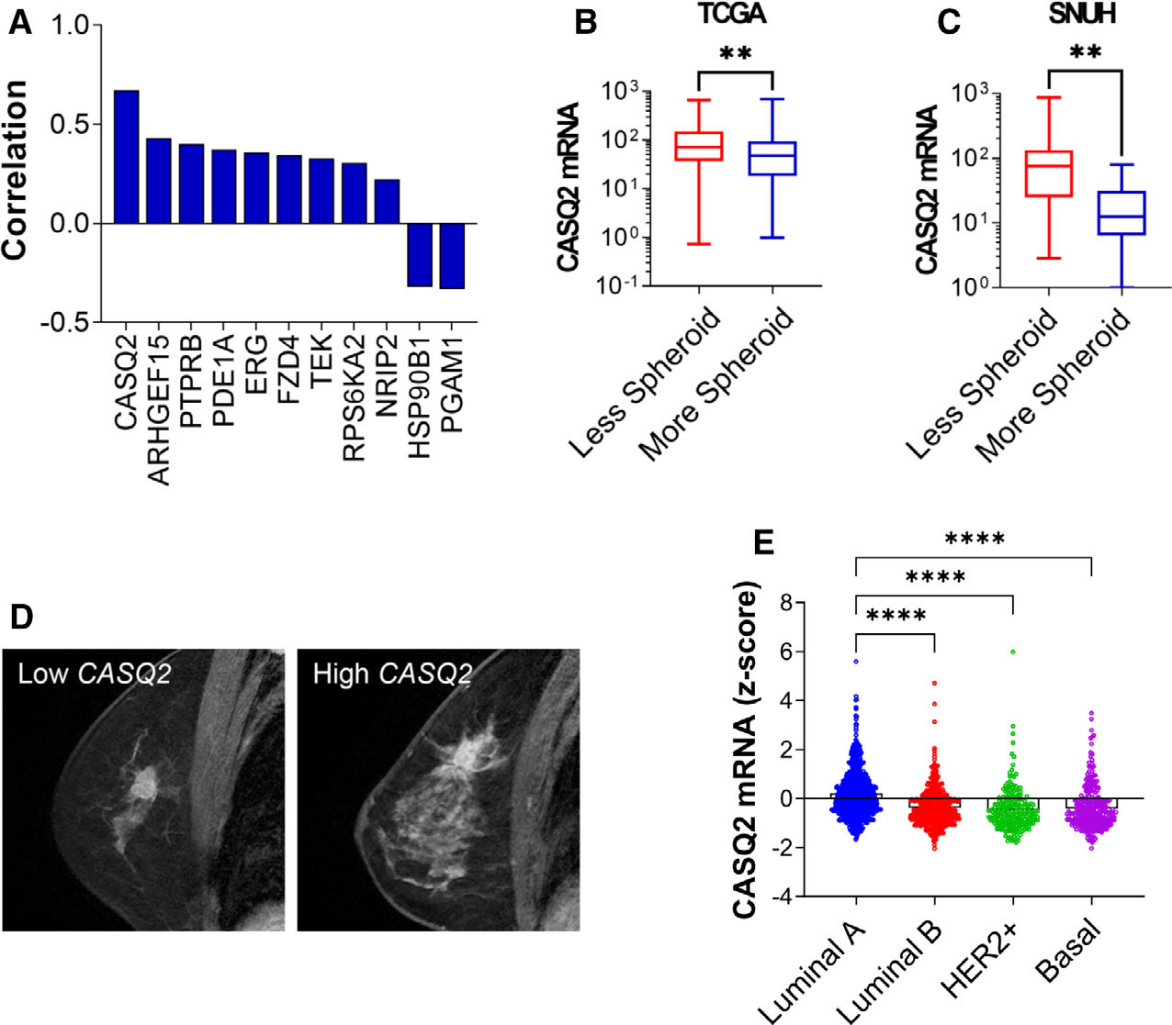
CASQ2 is a potential regulator of tumor–stroma interactions in breast cancer. (A) Positive correlation between the expression of The Cancer Genome Atlas (TCGA) breast cancer genes and spheroid–ellipsoid discrepancy (SED) value (*n* = 482; *P < *0.05 after the Benjamini–Hochberg correction). (B) Expression level of *CASQ2* mRNA in relation to tumor shape from TCGA dataset (*n* = 482; **P < 0.01 after the Mann–Whitney *U*‐test). (C) Expression level of *CASQ2* mRNA (RNA‐sequencing) according to the shape of tumors from Seoul National University Hospital (SNUH) patients (less spheroid, *n* = 22 and more spheroid, *n* = 10; **P < 0.01 after the Mann–Whitney *U*‐test). (D) Multiplanar reconstruction image of a magnetic resonance image. (E) Expression level of CASQ2 in breast cancer subtypes from the METABRIC dataset (*n* = 1640; ****P < 0.0001 after the Mann–Whitney *U*‐test).

We identified 482 cases in TCGA, for which three‐dimensional tumor diameters and mRNA expression data were available (Fig. S1). The degree of roundness was determined by measuring SED as previously reported [6]. By integrating the SED index and transcriptome data, we identified a set of genes exhibiting a significant correlation between their mRNA levels and the SED index (Fig. 1A). Calsequestrin 2 (*CASQ2*) showed the highest correlation with the SED index, indicating that tumors with a high expression of *CASQ2* exhibited more irregular and ellipsoid spatial shapes (Fig. 1A,B). Additionally, we obtained RNA‐sequencing data from 32 patients with breast cancer in our institution, for whom quantitative data on the degree of spatial roundness were available based on their preoperative magnetic resonance imaging. The expression of *CASQ2* was significantly upregulated in tumors with a high degree of irregularity (Fig. 1C,D).

We further explored the association between the expression level of *CASQ2* and breast cancer molecular subtypes as the spatial shape of breast cancer is related to the subtype [23]. Using the METABRIC database [24], we found that basal tumors had a similar expression of CASQ2 as luminal B and Her2 subtypes (Fig. 1E). The level of CASQ2 showed a modest association with tumor size, but not with nodal involvement and survival rate (Fig. S2A,B). We also analyzed the association between CASQ2 and clinicopathological variables in 482 cases of TCGA and found that the CASQ2 level significantly correlated with T stage, molecular subtype, and tumor shape (Table S1). In TCGA data, there was no significant difference in patient survival rate according to the level of CASQ2 and shape of the tumor (Fig. S2C). Additionally, we measured the CASQ2 mRNA level in the SNUH 143 patient cohort and observed that the high CASQ2 level is related to tumor irregularity in luminal A and basal type of breast cancer (Fig. S2D). Next, we performed the GSEA using TCGA data stratified with CASQ2 level and observed that the CASQ2 level was associated with calcium signaling, hematopoietic cell lineage, ECM receptor interaction, cell adhesion molecule, and cancer‐related pathways (Fig. S3). These results suggest that CASQ2 plays an important regulatory role in tumor growth, determining spatial tumor shape, and interaction with the TME. As TME interactions can be a determining factor of spatial tumor growth [7, 25, 26], and based on the above observations, we hypothesized that *CASQ2* could be a potential regulator of TME interaction in breast cancer.

### 3.2 *In vivo* induction of CASQ2 expression in breast cancer cells

**Fig. 2:**
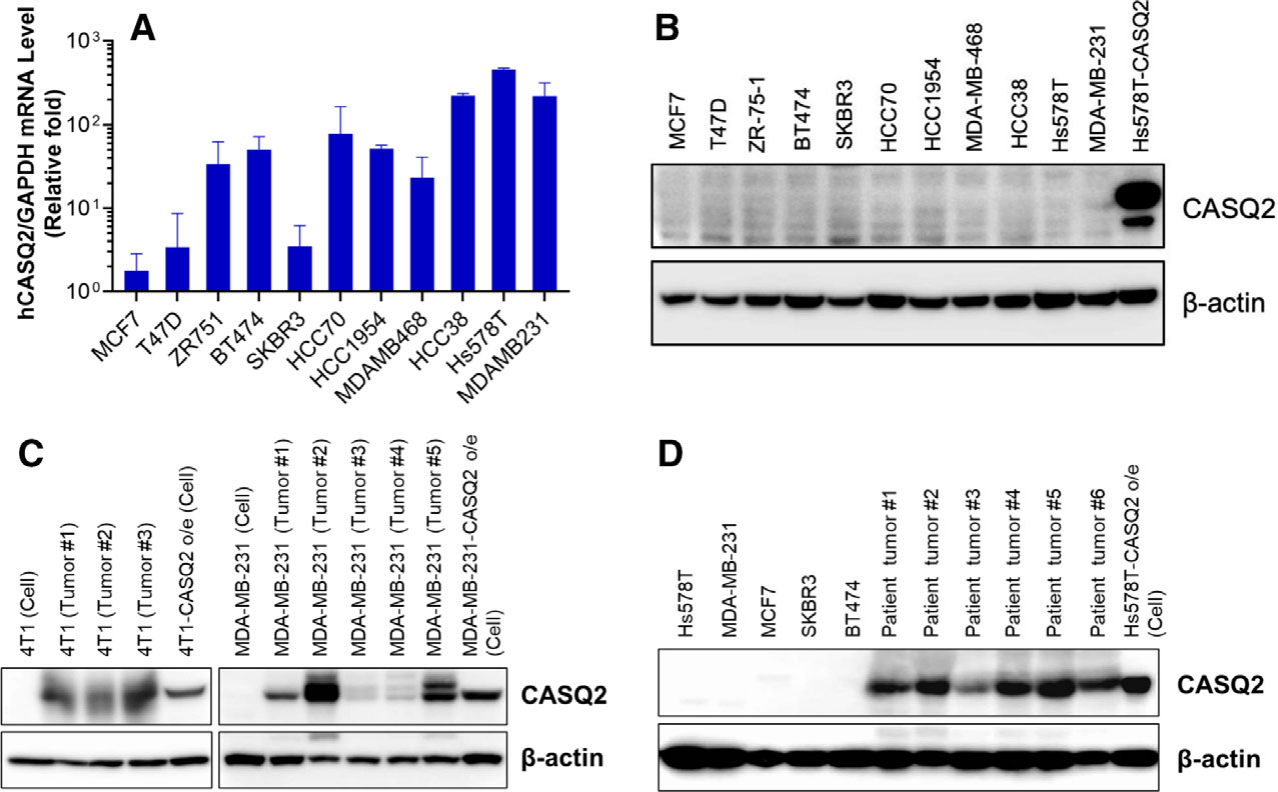
Expression of CASQ2 is induced in breast cancer tissues. (A) Level of *CASQ2* mRNA in human breast cancer cell lines. The experiment was repeated three times independently (mean ± SEM, *n* = 3). (B) Expression level of CASQ2 in breast cancer cell lines. (C) Expression level of CASQ2 in breast cancer cell lines and tumor tissues. (D) Protein expression in breast cancer cell lines and patient tumor tissues. All results (B–D) show one of three independent experiments (*n* = 3).

CASQ2 was originally identified as a cardiac muscle‐specific protein binding to Ca^2+^ of the sarcoplasmic reticulum, thereby regulating the intracellular Ca^2+^ concentration [27]. We evaluated the expression level of *CASQ2* in various breast cancer cell lines. Although various breast cancer cell lines displayed varying degrees of CASQ2 mRNA expression with relative fold changes (Fig. 2A), the actual mRNA expression levels were very low (data not shown), and we also did not determine the level of CASQ2 (Fig. 2B). Interestingly, when cancer cells were orthotopically injected into murine mammary fat pads, the resulting tumors were frequently positive for the expression of *CASQ2* (Fig. 2C, Fig. S4A,B). We found that CASQ2 was also expressed in fresh frozen‐resected breast cancer tissues (Fig. 2D).

**Fig. 3:**
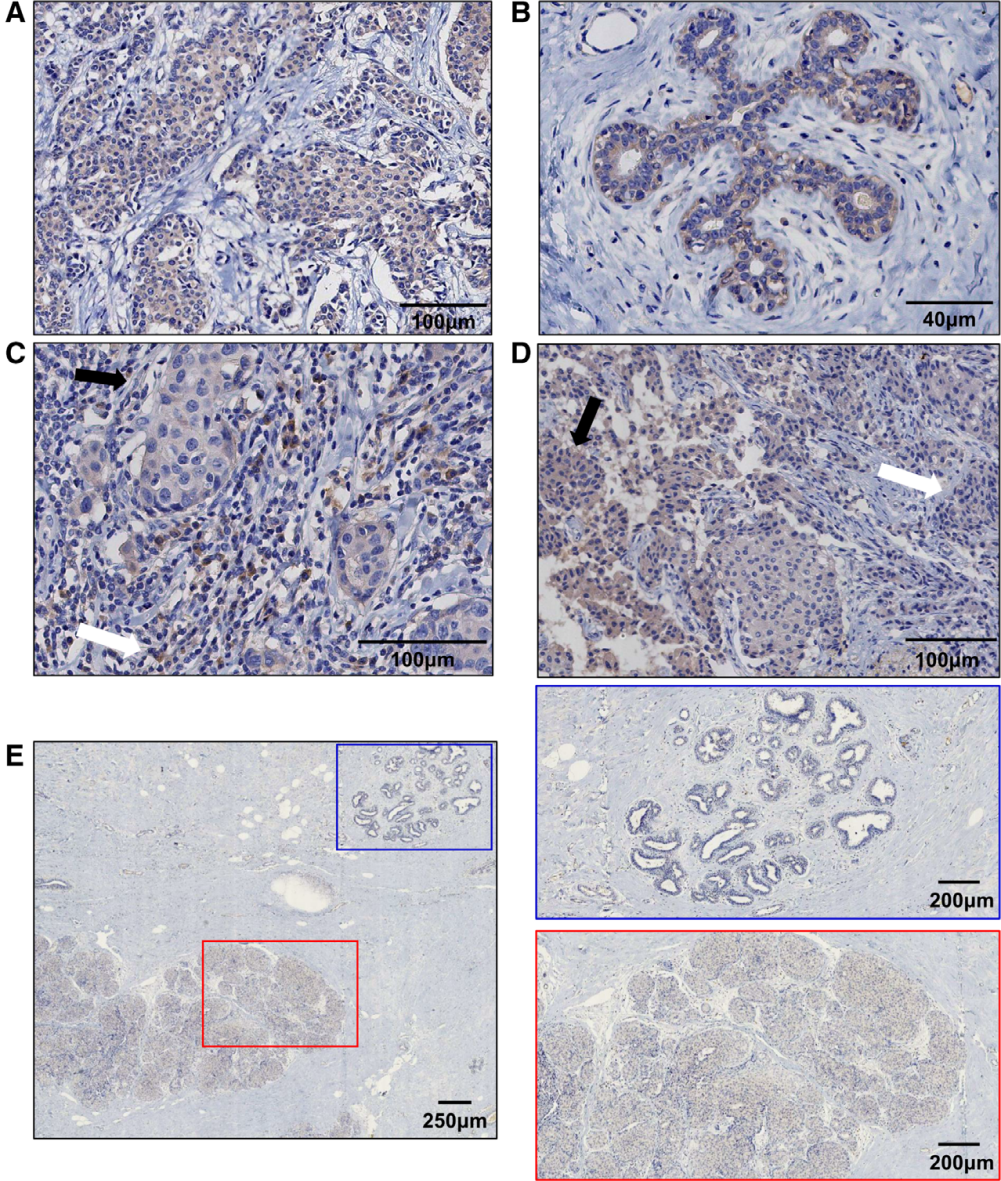
CASQ2 shows a heterogeneous expression pattern in breast cancer. Immunohistochemical analysis of the expression of CASQ2 in human breast cancer (A) and normal mammary epithelial (B) cells. (C) Expression of CASQ2 in stromal cells, including immune cells (white arrow) and fibroblasts (black arrow). (D) Heterogeneous spatial expression of CASQ2 in breast cancer tissues. (E) Positive and negative expression of CASQ2 in tumor (red box) and normal mammary epithelial (blue box) cells, respectively. Scale bars indicate 100 µm (A, C, D), 40 µm (B), 250 µm (E), and 200 µm (E). The figure shows one of three independent experiments.

Next, we performed immunohistochemical staining of human breast cancer tissues to determine the pattern of distribution of CASQ2. As shown in Fig. 3A,B, both human breast cancer cells and normal mammary epithelial cells expressed CASQ2. CASQ2 was also detected in stromal cells, such as immune cells and fibroblasts (Fig. 3C). Interestingly, breast cancer tissues exhibited a heterogeneous pattern of expression of CASQ2. More specifically, some tumors showed spatial heterogeneity in the expression of CASQ2 (Fig. 3D), whereas its expression was specifically induced in tumor cells but not in the adjacent normal mammary epithelial cells in other cases (Fig. 3E). As the *in vivo* microenvironment is known to induce substantial changes in the protein expression of many cancer cell lines [28, 29], this finding of the preferential expression of CASQ2 in TME *in vivo* suggested the involvement of this protein in tumor–stroma interactions during breast tumorigenesis.

### 3.3 CASQ2‐induced phenotypic changes in breast cancer cells

**Fig. 4:**
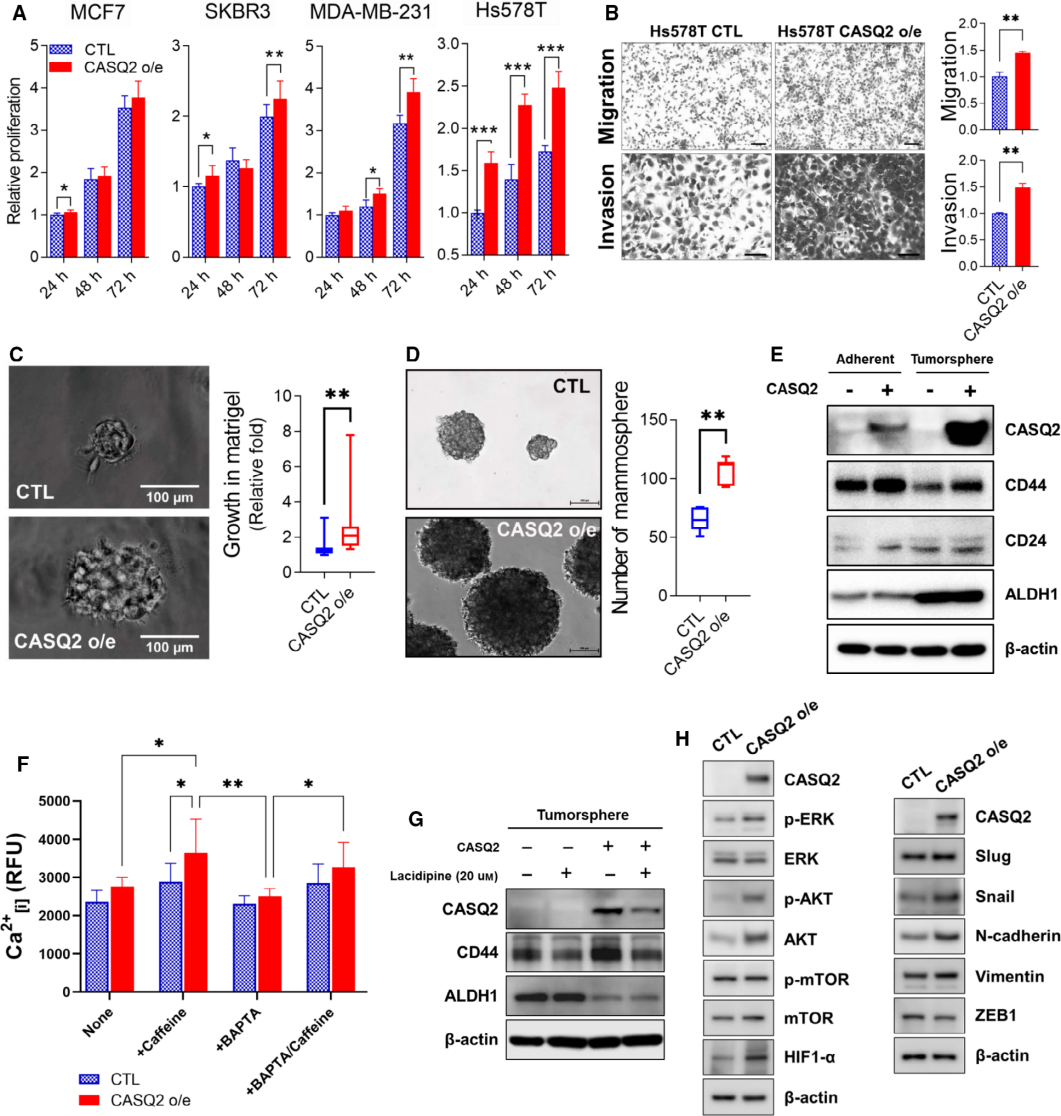
CASQ2 induces phenotypic changes in breast cancer cells. (A) Relationship between the proliferation rate and overexpression of CASQ2 in breast cancer cell lines (mean ± SEM, *n* = 3; *P < 0.05, **P < 0.01, and ***P < 0.001 using the multiple *t*‐test). (B) Migration and invasion rates of Hs578T cells (mean ± SEM, *n* = 3; **P < 0.01 using two‐tailed Student’s *t*‐test). Scale bar = 50 μm. (C) Three‐dimensional culture of Hs578T cells (mean ± SEM, *n* = 3; **P < 0.01 using two‐tailed Student’s *t*‐test). Scale bar = 100 μm. (D) Tumorsphere culture of Hs578T cells (mean ± SEM, *n* = 3; **P < 0.01 using two‐tailed Student’s *t*‐test). Scale bar = 100 μm. (E) Expression of CD44, CD24, and ALDH1 cancer stem cell markers in adherent and tumorsphere cultures of Hs578T cells. (F) Measurement of intracellular Ca^2+^ in Hs578T cells using a calcium crimson reagent. Cells were loaded with the calcium indicator calcium crimson (5 μm) for 30 min, and then, BAPTA‐AM (10 μm) was added. After washing, the cells were stimulated by treatment with 5 μm caffeine to measure the intracellular calcium concentration at 360 s (mean ± SEM, *n* = 3; *P < 0.05 and **P < 0.01 after ANOVA with Tukey’s *post hoc* test). (G) Effect of lacidipine on the expression of cancer stem cell markers in tumorspheres of breast cancer cells. The figure shows one of three independent experiments. Statistical test result is shown in Fig. S6. (H) Epithelial–mesenchymal transition (EMT)‐related protein expression in breast cancer cells. All results are representatives of three independent experiments.

To determine the functional importance of CASQ2 in breast cancer, we established various CASQ2‐overexpressing breast cancer cell lines (Fig. S5A). We specifically observed that the overexpression of CASQ2 did not induce major morphological changes in breast cancer cells (Fig. S5B), but promoted cell proliferation in multiple cell lines. This effect was observed in both MDA‐MB‐231 and Hs578T, with a higher effect in Hs578T, similar to the molecular characteristics of basal breast cancer subtype (Fig. 4A) [30]. In addition, the overexpression of CASQ2 resulted in slightly increased migration and invasion of Hs578T cells (Fig. 4B), but not other examined cell lines (Fig. S5C). In particular, when HS578T cells were cultured in a three‐dimensional Matrigel, overexpression of CASQ2 further increased their growth rate (Fig. 4C).

We also observed that the overexpression of CASQ2 resulted in enhanced mammosphere formation (Fig. 4D), which is a measure of cancer stem cell phenotype [31]. The expression of CASQ2 was further increased in mammosphere and was also associated with increased level of CD44, a well‐known marker of breast cancer stem cells (Fig. 4E) [32]. The overexpression of CASQ2 in breast cancer cells was also associated with increased intracellular calcium signaling. After caffeine treatment, the intracellular calcium concentration was significantly increased by CASQ2 overexpression, and it was confirmed that a higher concentration of calcium was maintained compared with the CTL even when caffeine was added after treatment with BAPTA, the calcium ion chelator (Fig. 4F). We next tested whether the concentration of intracellular calcium is related to the level of CD44. Consistent with the findings of a previous study [33], we observed that the CASQ2‐induced CD44 upregulation was inhibited by treatment with the calcium channel blocker, lacidipine, suggesting that the increase in the level of CD44 reflected the CASQ2‐induced changes in calcium signaling (Fig. 4G and Fig. S6). Notably, the expression of CASQ2 and ALDH1 (Fig. 4E), another breast cancer stem cell marker [34], was increased in the mammosphere culture condition.

Intracellular calcium signaling has also been associated with EMT in breast cancer cells [35]. Therefore, we tested the expression of various EMT‐related proteins in breast cancer cells and observed that the levels of several EMT markers, including slug, snail, and vimentin, were increased in CASQ2‐overexpressing cells and the level of ZEB1 was decreased (Fig. 4H). Moreover, we observed that overexpression of CASQ2 resulted in the upregulation of the expression of pAKT, AKT, and hypoxia‐inducible factor 1‐alpha (HIF1α) (Fig. 4H). Together, these findings indicated that CASQ2, a factor previously considered to be involved in cardiac functions, might also regulate various biological aspects of the breast cancer cell physiology and affect the cancer phenotype *in vitro*.

### 3.4 Overexpression of CASQ2 promotes tumorigenesis and ECM remodeling

**Fig. 5:**
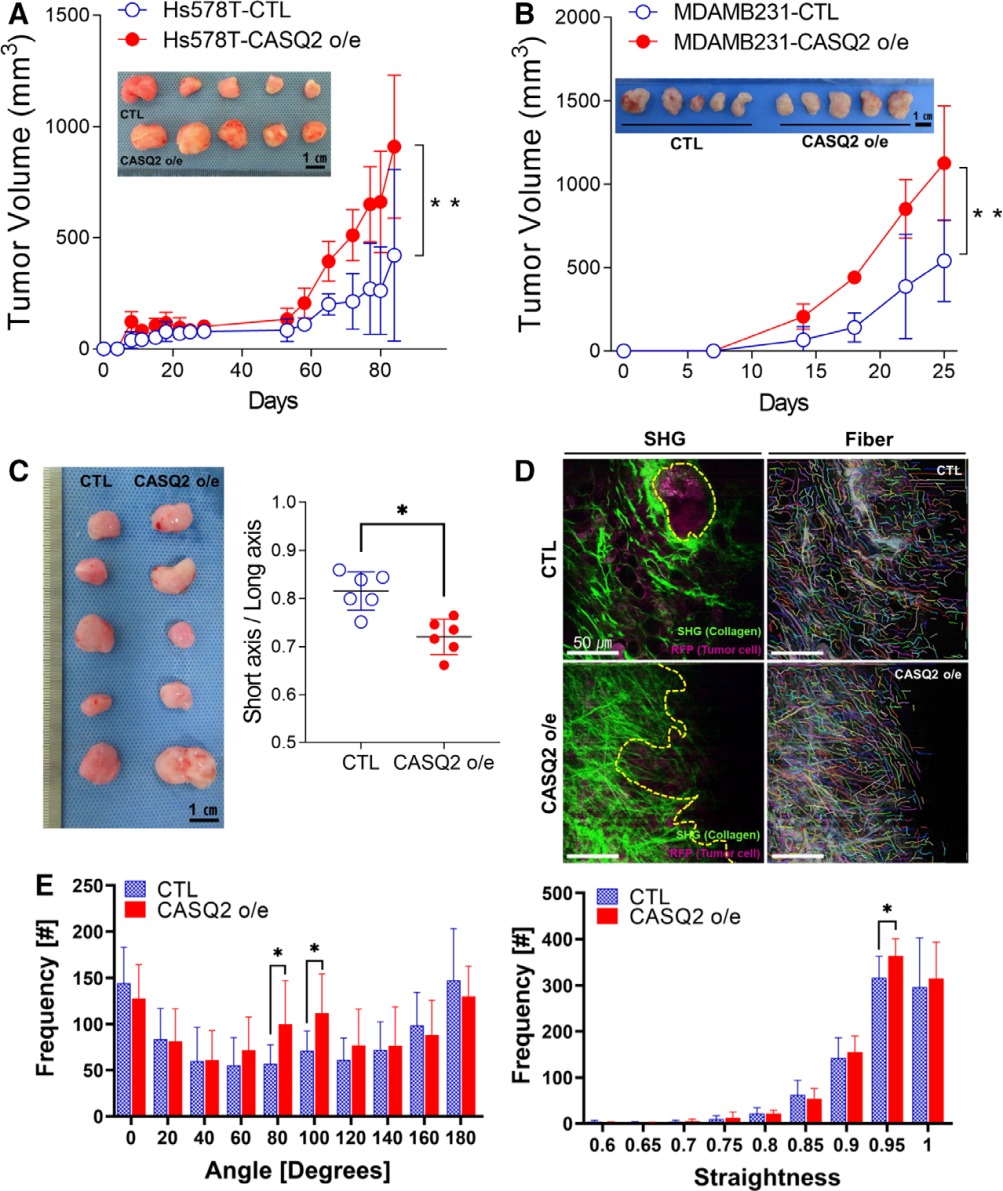
CASQ2 promotes tumorigenesis and ECM remodeling. Volume of tumors derived from (A) Hs578T and (B) MDA‐MB‐231 cells with or without CASQ2 o/e xenograft in NSG mice (mean ± SEM, *n* = 5; **P < 0.01 after a multiple *t*‐test). (C) Images of reimplanted tumor tissues and measurement of the spatial shape of tumor tissues (mean ± SEM, *n* = 5; *P < 0.05 after two‐tailed Student’s *t*‐test). (D) Second‐harmonic generation (SHG) imaging of collagen organization in xenograft Hs578T tumor tissues (red: tumor; green: collagen fiber; and yellow dotted line: collagen fiber angle). (E) The relative angle and straightness of collagen determined using fibers extracted in the CT‐FIRE mode (mean ± SEM, *n* = 15; *P < 0.05 after a multiple *t*‐test). Scale bars indicate 1 cm (A–C) and 50 µm (D).

To investigate the role of CASQ2 in breast cancer tumorigenesis, we injected control and CASQ2‐overexpressing Hs578T or MDA‐MB‐231 cells into the mammary fat pads of female NSG mice. We found that CASQ2‐overexpressing Hs578T and MDA‐MB‐231 cells exhibited significantly increased tumor growth rates compared with the control cells (Fig. 5A,B). As the level of CASQ2 mRNA showed a significant correlation with the spatial tumor shape, we examined the gross tumor shape in orthotopic mouse tumors. Although the tumors derived from the initial injection of cells showed no significant morphological differences (Fig. 5A), when the tumors were harvested and reimplanted in other mice, the CASQ2‐overexpressing Hs578T cells formed more elliptical tumors, whereas control cells formed more spheroid tumors (Fig. 5C). Furthermore, CASQ2‐overexpressing tumors were more proliferative through a high expression of Ki67 (Fig. S7). These data suggest that CASQ2 could promote *in vivo* tumor growth and might regulate the TME interaction during tumorigenesis.

Examination of the fibrillar collagen structures of the TME of xenograft tumors using second‐harmonic generation imaging [36] revealed no significant difference in the total amount of collagen (Fig. S8) but showed significantly increased frequency of straight‐angled collagen fibrillar structures in CASQ2‐overexpressing Hs578T tumors (Fig. 5D,E), which is an indicator of breast cancer invasiveness [37, 38, 39]. Together, these data suggest that CASQ2 promotes the growth of breast cancer and regulates various aspects of the TME, including the adjacent collagen structures and fibroblast education, potentially resulting in differences in spatial tumor growth.

### 3.5 Expression of CASQ2 was associated with HIF‐1 signaling and promotion of metastasis colonization

**Fig. 6:**
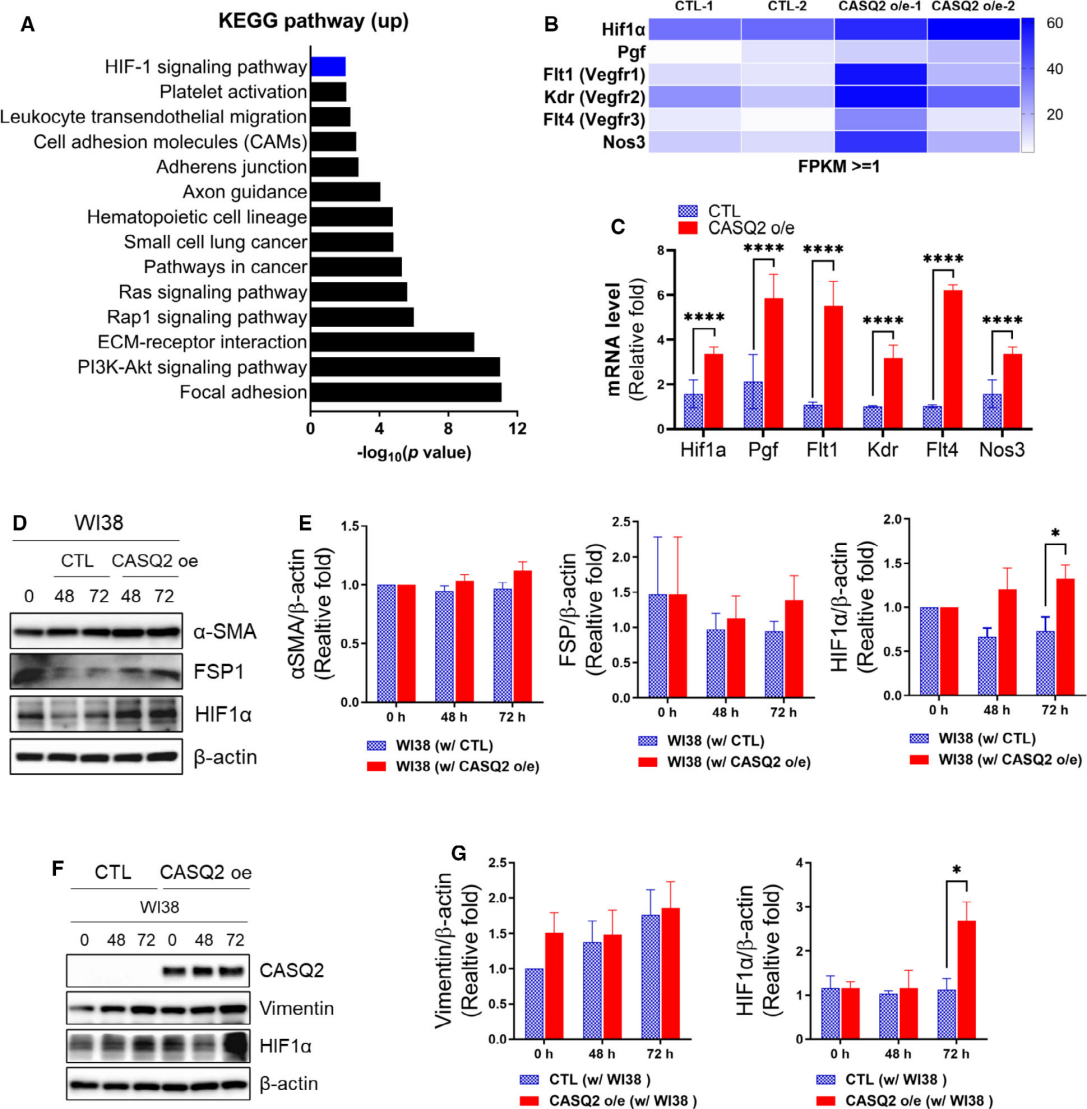
CASQ2 affects the TME and invasiveness through HIF1α. (A) Kyoto Encyclopedia of Genes and Genomes (KEGG) pathway analysis using the mouse genome (Differentially expressed genes, DEGs; *n* = 530). (B) The heatmap for average of expression (FPKM) value of most significant differentially expressed genes of angiogenesis and the HIF1α signaling pathway. (C) The mRNA level of Hif1a, Pgf, Flt1, Kdr, Flt4, and Nos3 in Hs578T‐CTL and CASQ2 o/e tumors (means ± SEM, *n* = 3; ***P < 0.001 by the multiple *t*‐test). (D, E) Expression level of α‐SMA, FSP, and HIF1α in WI‐38 cells after indirect coculture with Hs578T‐CTL or Hs578T‐CASQ2 o/e cells (means ± SEM, *n* = 3; *P < 0.05 by the multiple *t*‐test). (F, G) Expression level of CASQ2 and vimentin in Hs578T‐CTL or Hs578T‐CASQ2 o/e cells indirectly cocultured with WI‐38 (means ± SEM, *n* = 3; *P < 0.05 by the multiple *t*‐test).

**Fig. 7:**
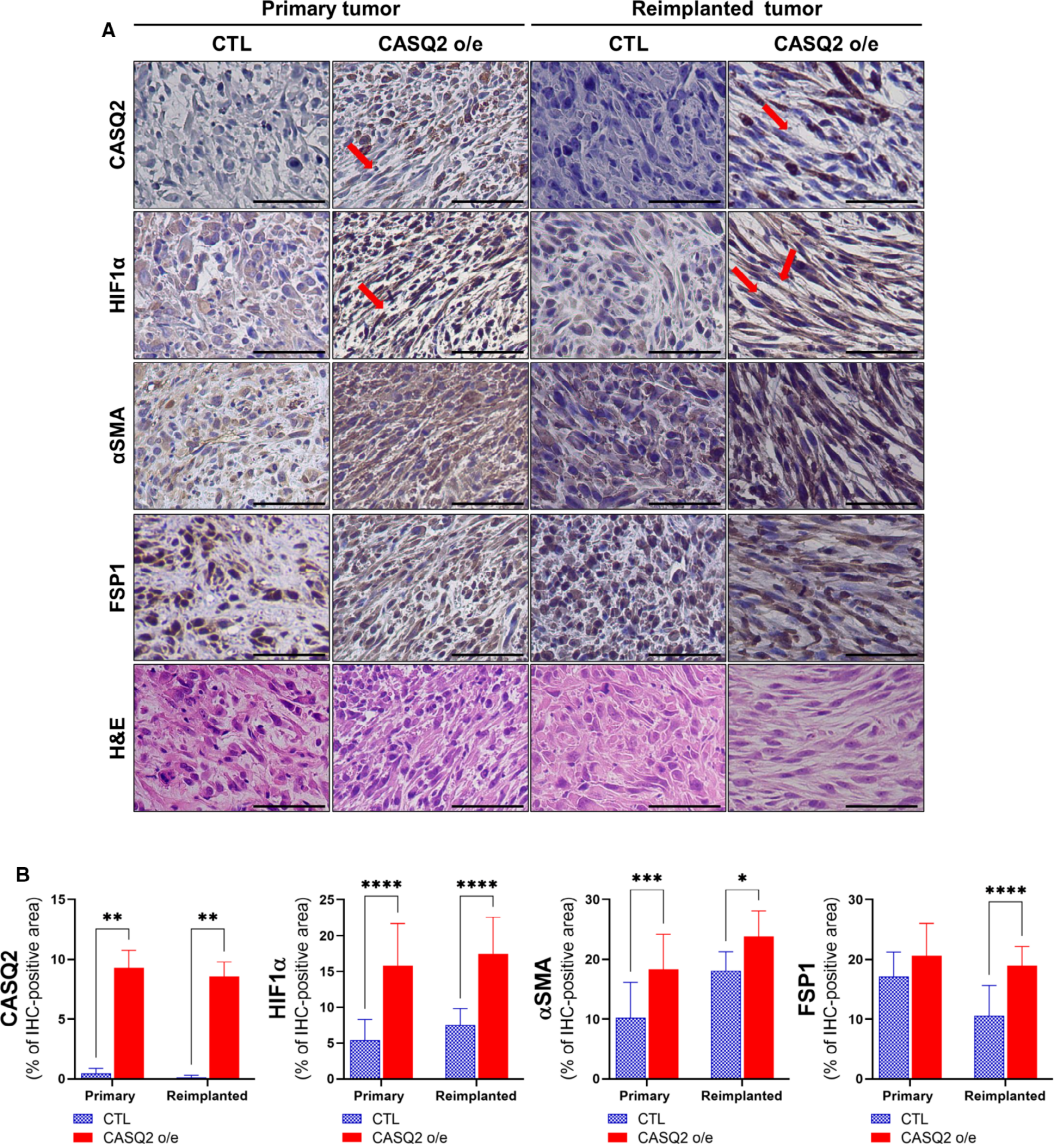
CASQ2 affects CAF and tumor shape through HIF1α. (A) Immunohistochemical images of HIF1α, α‐SMA, and FSP in Hs578T xenograft tumor tissues (red arrow; CAFs). (B) Staining density was measured and quantified with imagej (mean ± SEM, *n* = 5; *P < 0.05, **P < 0.01, ***P < 0.001, and ****P < 0.0001 by two‐tailed Student’s *t*‐test). Scale bar = 100 µm.

RNA sequencing of xenograft tumors can also provide insights into the changes in the expression of the murine TME [40]. Therefore, we separately analyzed murine RNA‐sequencing reads and observed that several signaling pathways were dysregulated in CASQ2‐overexpressing Hs578T tumors. Notably, we found that genes involved in HIF‐1 signaling pathways were significantly altered within the murine TME (Fig. 6A). The RNA‐sequencing data and qRT‐PCR results showed that the HIF1α and HIF1α signaling‐related genes (*Pgf*, *Flt1*, *Kdr*, *Flt4*, and *Nos3*) were significantly upregulated in CASQ2 o/e tumors (Fig. 6B,C). To determine whether HIF1α expression in the tumor microenvironment is related to CASQ2 overexpression, we checked HIF1α and cancer‐associated fibroblast markers [41, 42], FSP1 and αSMA, after indirect culture with stromal WI‐38 fibroblasts. In WI‐38 fibroblasts, there was significant upregulation of HIF1α when the cells were cocultured with the CASQ2 o/e cancer cells (Fig. 6D,E). Although the mRNA level of αSMA was significantly increased (Fig. S9A), the protein levels of αSMA and FSP1 did not show such a difference (Fig. 6D,E). Interestingly, HIF1α upregulation was also observed in the cocultured CASQ2 o/e Hs578T cells suggesting a potential HIF1α‐mediated interaction (Fig. 6F,G and Fig. S9B). Next, we investigated the levels of HIF1α and CAF markers in primary and serially transplanted Hs578T xenograft tumors. As shown in Fig. 7A,B, the expression of HIF1α, αSMA, and FSP1 in CASQ2 o/e tumors was significantly higher than that in CTL tumors, in both primary and serially transplanted tumors. The serially transplanted tumor exhibited more spindle‐like tumor cells and higher expression of αSMA and FSP1 in the fibroblasts around the CASQ2‐expressing cancer cells. This phenomenon was more pronounced in CASQ2 o/e tumors than in CTLs. These results suggest that CASQ2 expression may induce features of CAF adjacent to tumor cells.

**Fig. 8:**
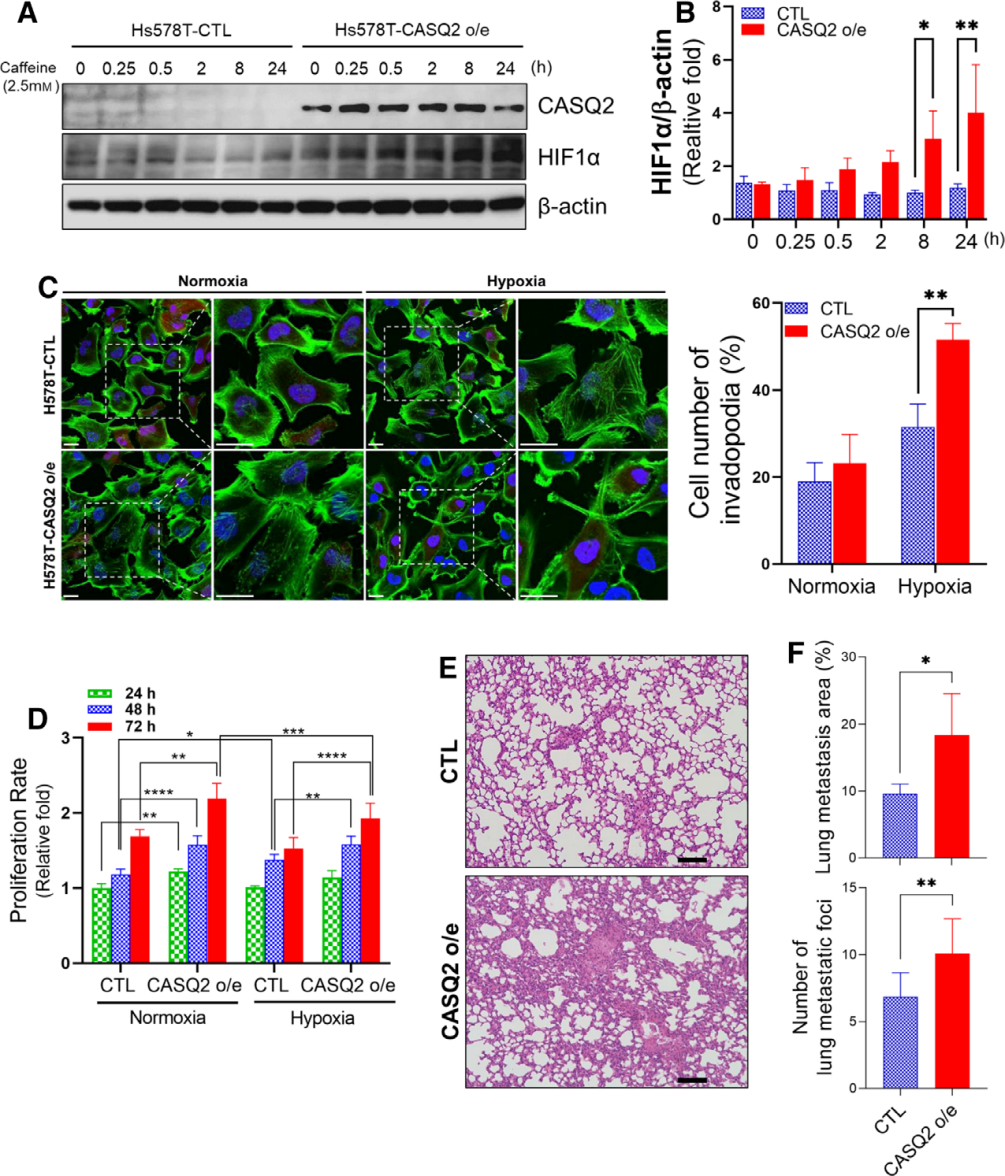
CASQ2 affects the tumor invasiveness through HIF1α. (A, B) Expression level of HIF1α in caffeine‐treated Hs578T cells with or without overexpression of CASQ2 (means ± SEM, *n* = 3; *P < 0.05, **P < 0.01 by the Mann–Whitney *U*‐test). (C) Confocal microscopy images showing F‐actin in Hs578T cells overexpressing CASQ2 under hypoxic conditions (**P < 0.01 by the Mann–Whitney *U*‐test). Scale bar = 10 μm. (D) Proliferation rate of control (CTL) and CASQ2‐overexpressing (CASQ2 o/e) breast cancer cells under normoxic and hypoxic conditions (mean ± SEM, *n* = 3; *P < 0.05, **P < 0.01, ***P < 0.001, and ****P < 0.0001 by ANOVA with Tukey’s *post hoc* test). (E, F) Hematoxylin and eosin staining of lung tissue sections from Hs578T‐CTL‐ or CASQ2‐derived tumors (mean ± SEM, *n* = 5; *P < 0.05 and **P < 0.01 by two‐tailed Student’s *t*‐test). Scale bar = 100 μm.

The overexpression of CASQ2 resulted in the upregulation of the baseline and calcium‐induced protein levels of HIF1α in Hs578T cells (Fig. 8A,B). Based on these results, we tested whether the overexpression of CASQ2 might affect the phenotype of cancer cells under hypoxia using a hypoxic chamber *in vitro*. We observed that control Hs578T cells displayed an epithelial‐like morphology under both normoxic and hypoxic conditions. However, CASQ2‐overexpressing cells were found to exhibit significant morphological changes toward more spindle‐like shapes and a higher incidence of invadopodia‐like structures, which is a morphological change in cancer cells during migration and invasion by an increase in HIF1α expression under hypoxic conditions [43] (Fig. 8C). However, although CASQ2‐overexpressing cells had a higher proliferation rate than CTLs under normoxia and hypoxia, this phenomenon did not induce significant differences following hypoxia (Fig. 8D). Hypoxia and subsequent invadopodia formation have been reported to play a critical role in the development of distant metastasis of solid tumors [44]. To investigate the effect of CASQ2 on breast cancer metastasis, we injected cells into the tail vein of NSG mice. We found that CASQ2‐overexpressing cells showed significantly higher metastatic areas and foci numbers than control cells (Fig. 8E,F). Together, these data indicated that CASQ2 regulates the transition of the hypoxia‐induced phenotype in breast cancer cells, supporting its role in the process of distant metastasis.

### 3.6 CASQ2 modulated the features of human metaplastic carcinoma in breast cancer cells

Metaplastic breast cancer is a subtype of breast cancer that is characterized by unique features, such as a spindle‐like mesenchymal morphology, the capacity to differentiate into sarcomatous components, and marked resistance to therapeutics [45]. Hs578T breast cancer cell line is often suggested to be a metaplastic carcinoma cell line as it was originally derived from a tumor with a spindle‐shaped sarcomatous feature [46] and has been genomically classified as a mesenchymal‐like basal B subtype [47]. As CASQ2 was demonstrated to cause the most distinct phenotypic changes in Hs578T cells among several breast cancer cell lines examined, we hypothesized that the expression of CASQ2 could be associated with the features of human metaplastic carcinoma.

**Fig. 9:**
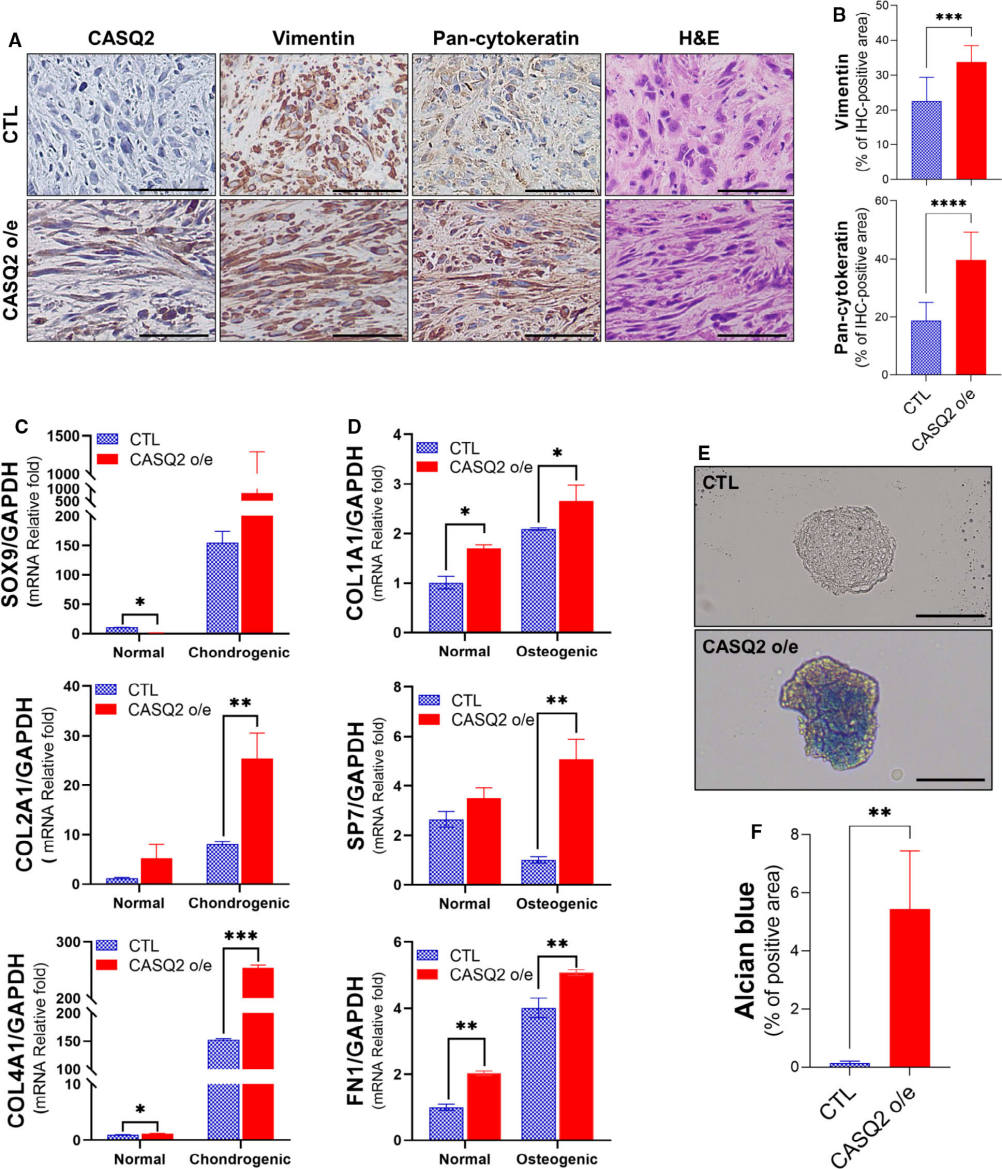
CASQ2 is an important modulator of the development of metaplastic carcinoma. (A, B) Immunohistochemical images of pan‐cytokeratin and vimentin in Hs578T xenograft tumor tissues (mean ± SEM, *n* = 5; ***P < 0.001 and ****P < 0.0001 by two‐tailed Student’s *t*‐test). The experiment was repeated three times independently. (C) mRNA levels of chondrogenesis marker genes in Hs578T‐CTL and Hs578T‐CASQ2 o/e cells (mean ± SEM, *n* = 3; *P < 0.05, **P < 0.01, and ***P < 0.001 by ANOVA with Tukey’s *post hoc* test). (D) mRNA levels of osteogenesis markers after osteogenic differentiation in Hs578T‐CTL and Hs578T‐CASQ2 o/e cells (mean ± SEM, *n* = 3; *P < 0.05 and **P < 0.01 by ANOVA with Tukey’s *post hoc* test). (E, F) Alcian blue staining after chondrogenic differentiation of Hs578T‐CTL and Hs578T‐CASQ2 o/e cells (mean ± SEM, *n* = 3; **P < 0.01 by two‐tailed Student’s *t*‐test). Scale bar = 100 μm. All results are representatives of three independent experiments.

We found that CASQ2‐overexpressing Hs578T cells displayed substantial morphological changes *in vivo*. Similarly, CASQ2‐overexpressing tumors contain more mesenchymal and spindle‐shaped tumor cells compared with control tumors (Fig. 9A,B). The histological diagnostic criteria of metaplastic carcinoma include a spindle‐like morphology, and dual expression of cytokeratin and vimentin [48]. However, we observed that despite their fibroblast‐like morphology, CASQ2‐overexpressing tumor cells still showed positive expression of cytokeratin, suggesting an epithelial lineage (Fig. 9A,B). Moreover, CASQ2‐overexpressing tumor cells showed a stronger expression of vimentin than the control cells (Fig. 9A,B).

Human metaplastic carcinoma often displays histological features of mesenchymal differentiation, such as chondrogenic and osteogenic differentiation [16]. When cells were cultured under chondrogenic or osteogenic differentiation conditions, the expression of CASQ2 was further upregulated in CASQ2‐overexpressing Hs578T cells (Fig. 9C,D). We then measured the mRNA levels of chondrogenesis markers such as *SOX9*, *COL2A1*, and *COL4A* and observed an increase in their levels, especially in CASQ2 o/e cells (Fig. 9C). We also confirmed the increase in osteogenic markers *COL1A1*, *SP7*, and *FN1* under osteogenic conditions (Fig. 9D). We observed that both culture conditions often induced the expression of chondrogenic and osteogenic markers in control Hs578T cells. However, the upregulation of both markers was demonstrated to be significantly enhanced in CASQ2‐overexpressing cells. Furthermore, we found that CASQ2‐overexpressing Hs578T cells showed positive staining for Alcian blue, a marker for cartilage [49], under chondrogenic differentiation culture conditions (Fig. 9E,F). Together, these data indicated that CASQ2 might be a major regulator of breast cancer differentiation toward metaplastic features.

## 4 Discussion

In this study, we identified *CASQ2* as an important gene regulating various aspects of breast cancer progression. CASQ2 is the main Ca^2+^‐binding protein in the sarcoplasmic reticulum of cardiomyocytes, forming a complex with ryanodine receptor 2, a luminal calcium channel of the cardiac muscle [50]. Although CASQ2 has been associated with catecholaminergic polymorphic ventricular tachycardia, its role in breast cancer has not been reported before.

Our data show that CASQ2 can promote the growth and metastasis of breast cancer cells. Although CASQ2 was expressed at a considerably low level in two‐dimensional cell culture condition, we observed a significant upregulation of CASQ2 during *in vivo* growth or tumorsphere culture condition. Additionally, in line with the findings of recent studies, which showed that calcium signaling can be a major regulator of cancer stem cell on solid tumors [51, 52], our findings suggest that CASQ2‐overexpressing cells show several surrogate features of cancer stem cells, such as tumorsphere growth and CD44 expression upregulation, which was reversed by lacidipine treatment. Indeed, previous studies have documented the potential involvement of calcium signaling in CD44^+^ breast cancer cells [53, 54]. Our findings suggest that breast cancer cells may undergo dynamic changes in calcium signaling *in vivo* via the induction of CASQ2, which may in turn regulate the stem cell properties during tumor growth and metastasis.

The biological mechanisms underlying the spatial growth pattern in solid tumor are unknown. One possible explanation is that the spatial tumor shape can be influenced by the tumor and microenvironment interactions [18]. Interestingly, we observed that CASQ2 activated the features of cancer‐associated fibroblasts in the cocultured fibroblasts. Additionally, CASQ2 upregulated HIF1α expression in both tumor cells and cocultured fibroblasts. CASQ2 further affected the patterns of collagen rearrangement within the microenvironment, which has been linked to the more aggressive features of breast cancer [37]. These data indicate that CASQ2 remodels various components of tumor microenvironment including CAF activation and hypoxia signaling in fibroblasts and remodeling of extracellular matrix.

Metaplastic carcinoma of breast is a rare entity of breast malignancy with poor treatment outcomes [55]. CASQ2 overexpression resulted in more mesenchymal morphological features and upregulation of vimentin expression, which are the characteristics of metaplastic carcinoma. Furthermore, CASQ2 upregulated genes associated with mesenchymal cell differentiation, which is another feature of metaplastic carcinoma. However, we were not able to elucidate the molecular functions of CASQ2 in metaplastic carcinoma and demonstrate the clinical significance of CASQ2 in human metaplastic carcinoma samples, and these are the major limitations of the present study.

Additionally, our *in vitro* data suggest that CASQ2 may have different biological significance in various breast cancer cell lines suggesting the cell type‐specific function of CASQ2. We were not able to elucidate the molecular mechanisms of how CASQ2 expression is only activated *in vivo*. Finally, while some of the CASQ2‐induced features were reversed with calcium channel inhibitors, it is not clear whether the effect of CASQ2 is dependent on calcium signaling or on other noncanonical functions of CASQ2. Several studies have shown that calcium‐regulating factors such as TRPV1, SERCA, ORAI, and STIM affect cancer metastasis and growth [56]. However, the overexpression of CASQ2 did not affect the expression of other calcium‐regulating genes in this study (data not shown).

## 5 Conclusion

We showed that CASQ2, a gene previously considered to be involved in cardiac function, is an important regulator of breast cancer progression and metastasis. CASQ2 also mediates various aspects of TME interactions in breast cancer. The role of CASQ2 in human breast cancers, as well as the influence of calcium modulation on the effect of anticancer agents, should be further explored.

## Conflict of interest

The authors declare no conflict of interest.

## Author contributions

JHK contributed to experimental design, conduction of experiment conduction, data analysis, funding, and manuscript preparation. E‐SL, HSR, and HKK performed data analysis, patient management, and histological studies. YWJ provided assistance with the experiments. JY and KK performed statistical analysis and computational analysis and provided feedback on experimental design. JIK was involved in the conception of the research and experimental design. H‐GM was involved in the conception of the research, experimental design, data analysis, funding, and manuscript preparation. All authors read and approved the final manuscript.

### Peer Review

The peer review history for this article is available at https://publons.com/publon/10.1002/1878‐0261.13136.

## Supporting information


**Fig. S1**. Gene analysis and workflow.
**Fig. S2**. CASQ2 expression and spatial shape analysis in METABRIC, TCGA, and SNUH cohort.
**Fig. S3**. GSEA of TCGA BRCA stratified with CASQ2 expression levels.
**Fig. S4**. Immunohistochemical staining of CASQ2 and H&E staining of breast tumor cell xenograft model.
**Fig. S5**. CASQ2‐induced phenotypic changes in breast cancer cells.
**Fig. S6**. Effect of lacidipine on the expression of cancer stem cell markers in tumorspheres of breast cancer cells.
**Fig. S7**. Immunohistochemical images of Ki67 in Hs578T xenograft tumor tissues.
**Fig. S8**. SHG+ collagen density in Hs578T‐CTL and Hs578T‐CASQ2 o/e tumor tissues.
**Fig. S9**. CASQ2 mediates the conversion of stromal cells to cancer‐associated fibroblasts.

**Table S1**. Proportions of patients with low and high CASQ2 mRNA levels according to clinicopathologic parameters of TCGA dataset.

## Data Availability

All data generated or analyzed during this study are included in this published article and its Supporting Information files.
