# Supplementary material for: Calsequestrin 2 overexpression in breast cancer increases tumorigenesis and metastasis by modulating the tumor microenvironment
Source: Mol Oncol. 2021 Nov 14;16(2):466–84. doi: 10.1002/1878-0261.13136 (PMC8763655; doi:10.1002/1878-0261.13136)
Supplement: Supplementary file 1 — Fig. S1. Gene analysis and workflow. Fig. S2. CASQ2 expression and spatial shape analysis in METABRIC, TCGA, and SNUH cohort. Fig. S3. GSEA of TCGA BRCA stratified with CASQ2 expression levels. Fig. S4. Immunohistochemical staining of CASQ2 and H&E staining of breast tumor cell xenograft model. Fig. S5. CASQ2‐induced phenotypic changes in breast cancer cells. Fig. S6. Effect of lacidipine on the expression of cancer stem cell markers in tumorspheres of breast cancer cells. Fig. S7. Immunohistochemical images of Ki67 in Hs578T xenograft tumor tissues. Fig. S8. SHG+ collagen density in Hs578T‐CTL and Hs578T‐CASQ2 o/e tumor tissues. Fig. S9. CASQ2 mediates the conversion of stromal cells to cancer‐associated fibroblasts. [file MOL2-16-466-s002.docx]

**Roles of calsequestrin 2 on tumor microenvironment, tumorigenesis, and metastasis of breast cancer**

**Supplementary Information**

**
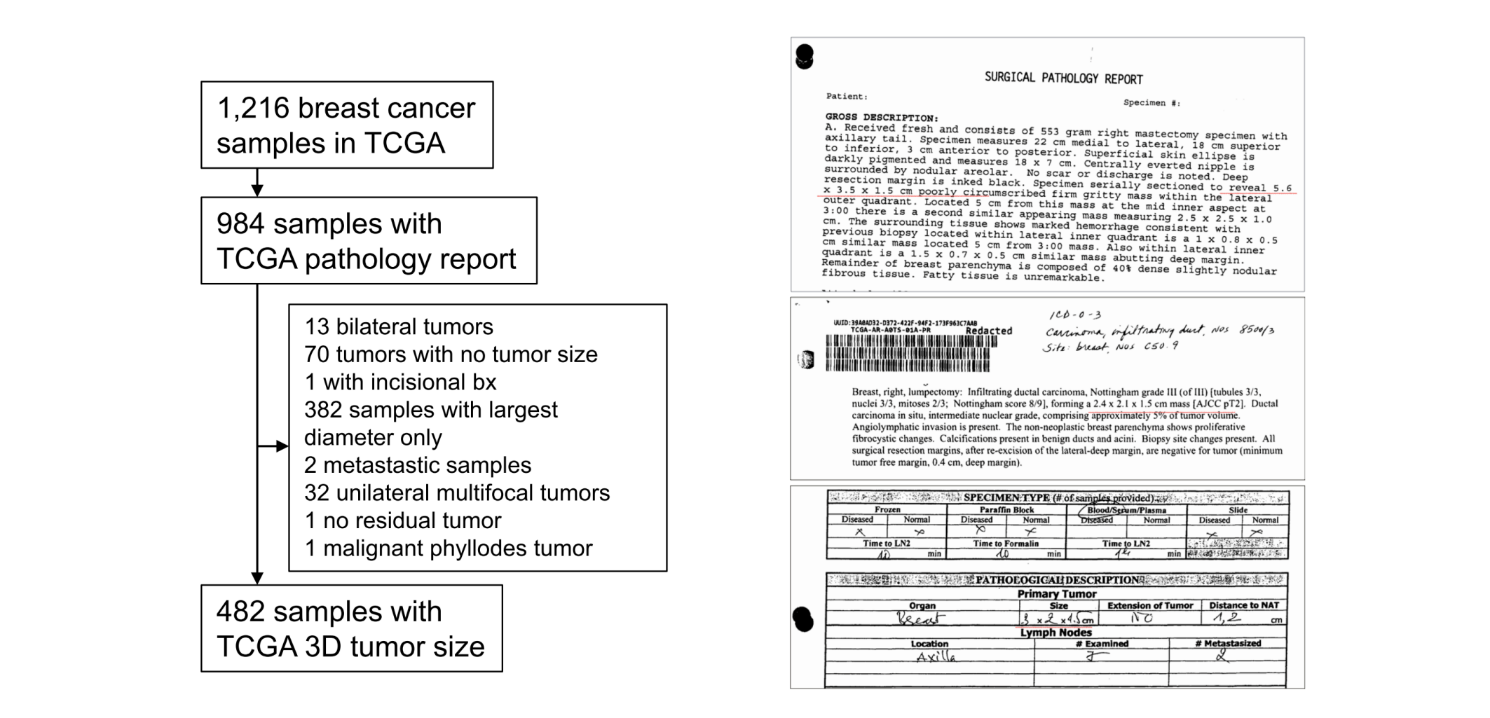
**

**Supplementary Figure S1. Gene analysis and workflow.**


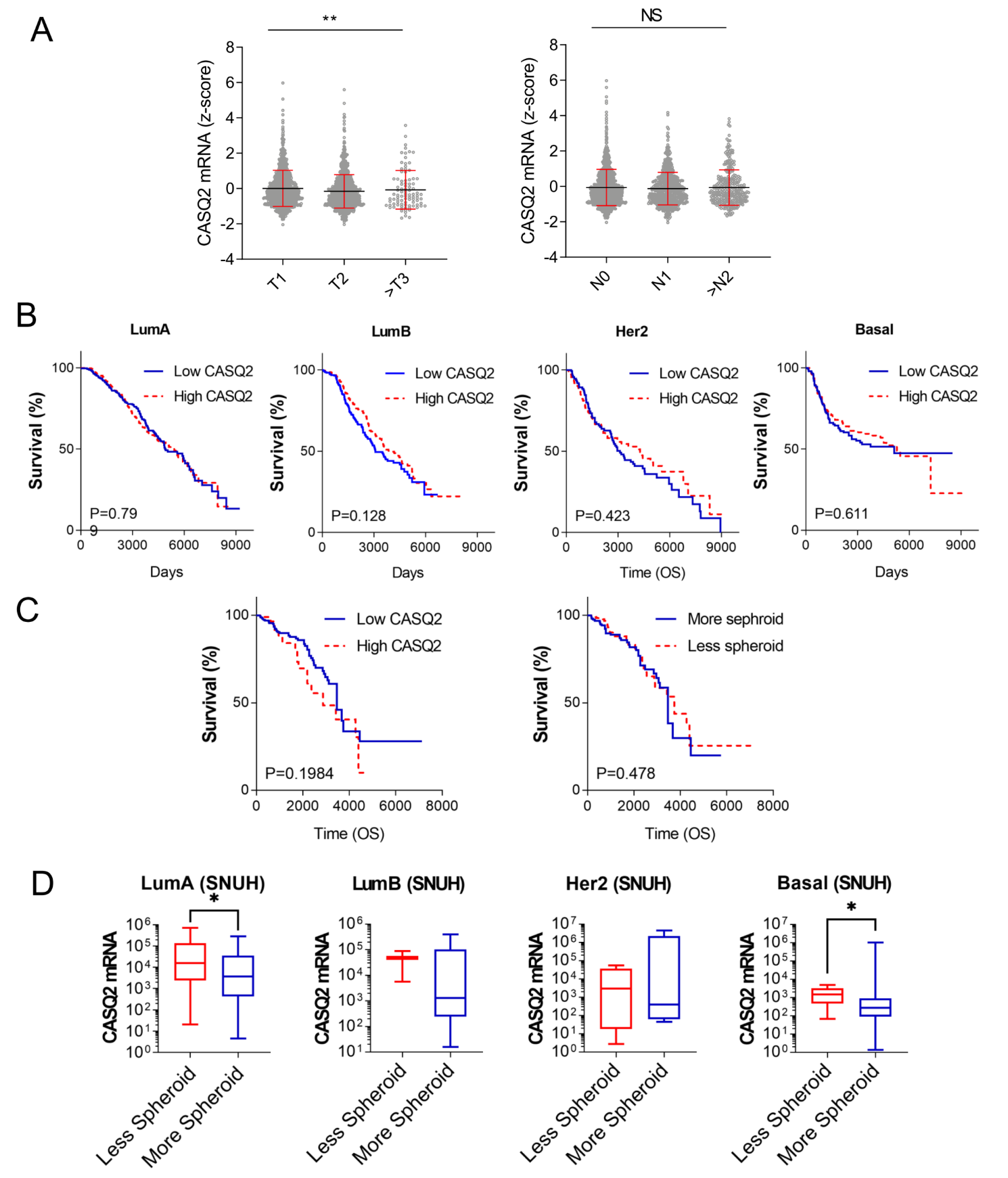


**Supplementary Figure S2.** **CASQ2 expression and spatial shape analysis in the METABRIC, TCGA, and SNUH cohort.** (A) CASQ2 expression level according to tumor size and nodal stage in breast cancer from the METABRIC datasets (*^**^p* < 0.01 by one-way ANOVA). (B) Kaplan–Meier curves with median from METABRIC RNA-seq (n = 1108, two-sided log-rank test). (C) Kaplan–Meier curves from TCGA (n = 482, two-sided log-rank test). (D) Expression level of *CASQ2* mRNA according to the shape of tumors from 143 Seoul National University Hospital (SNUH) patients (*^*^p* < 0.05 by Mann–Whitney *U* test)


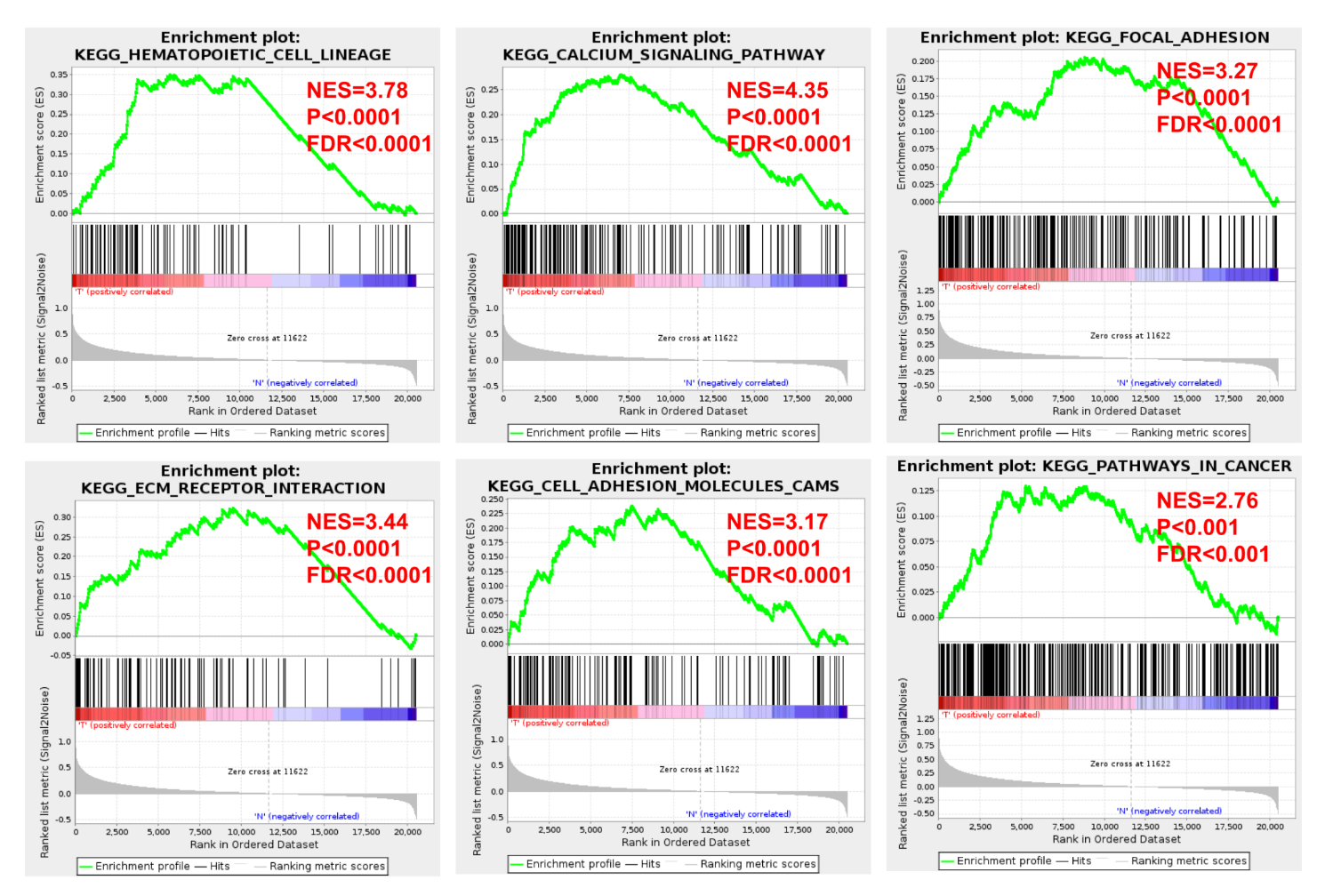


**Figure S3.** **GSEA of TCGA BRCA stratified by CASQ2 expression levels.**


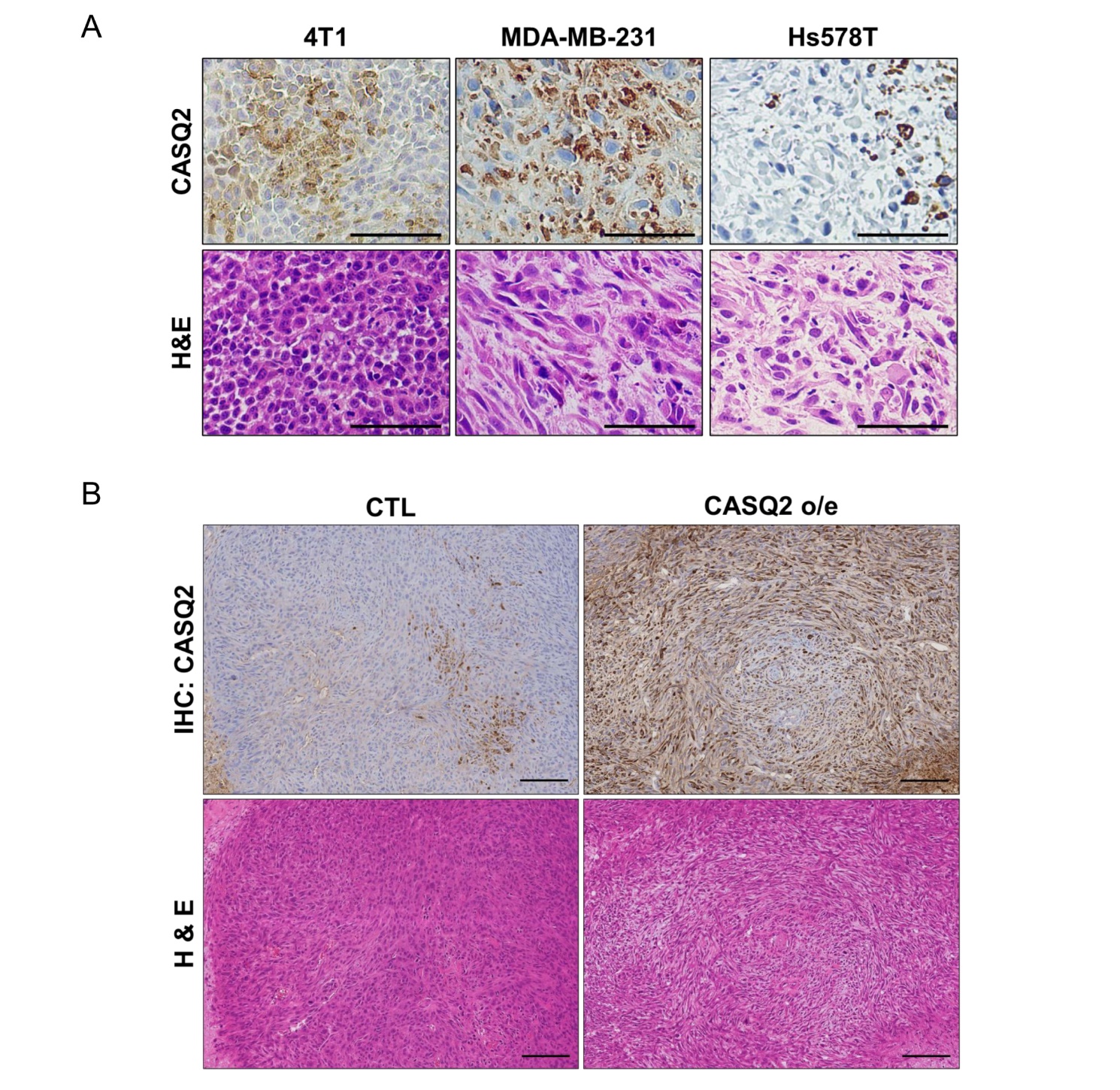


**Figure S4. Immunohistochemical staining of CASQ2 and H&E staining of breast tumor cell xenograft model.** (A) Tissues from 4T1, MDA-MB-231, and Hs578T xenograft tumor. (B) Tissues from Hs578T-CTL and Hs578T-CASQ2 o/e xenograft tumor. Scale bar = 100 µm.


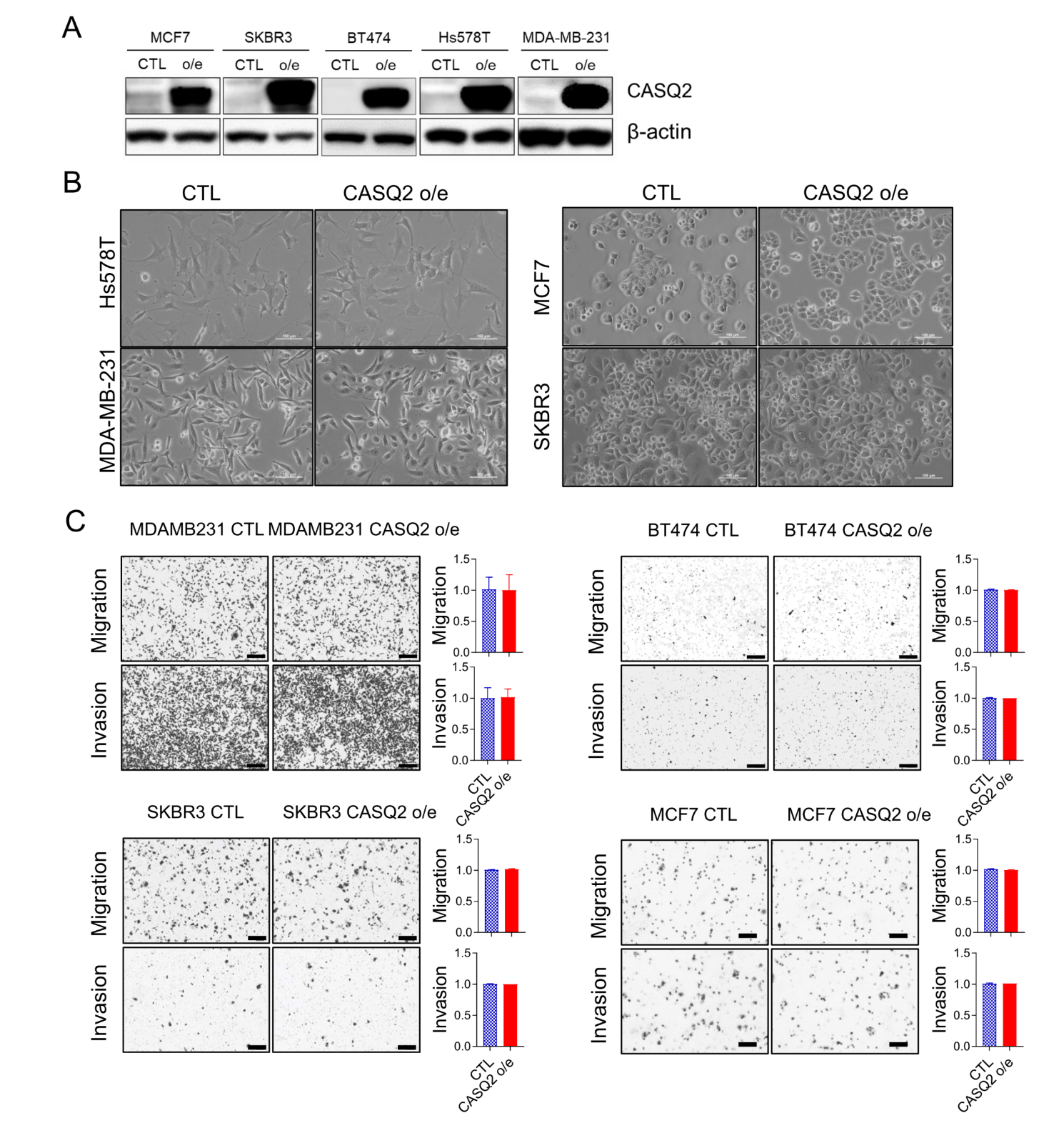


**Supplementary Figure S5. CASQ2-induced phenotypic changes in breast cancer cells.** (A) Level of CASQ2 overexpression in breast cancer cell lines. (B) Representative morphological changes in CASQ2-overexpressing breast cancer cell lines. (C) Migration and invasion of breast cancer cell lines with or without CASQ2 overexpression (means ± SEM, n = 5; **p* < 0.05 by two-tailed Student’s *t*-test).


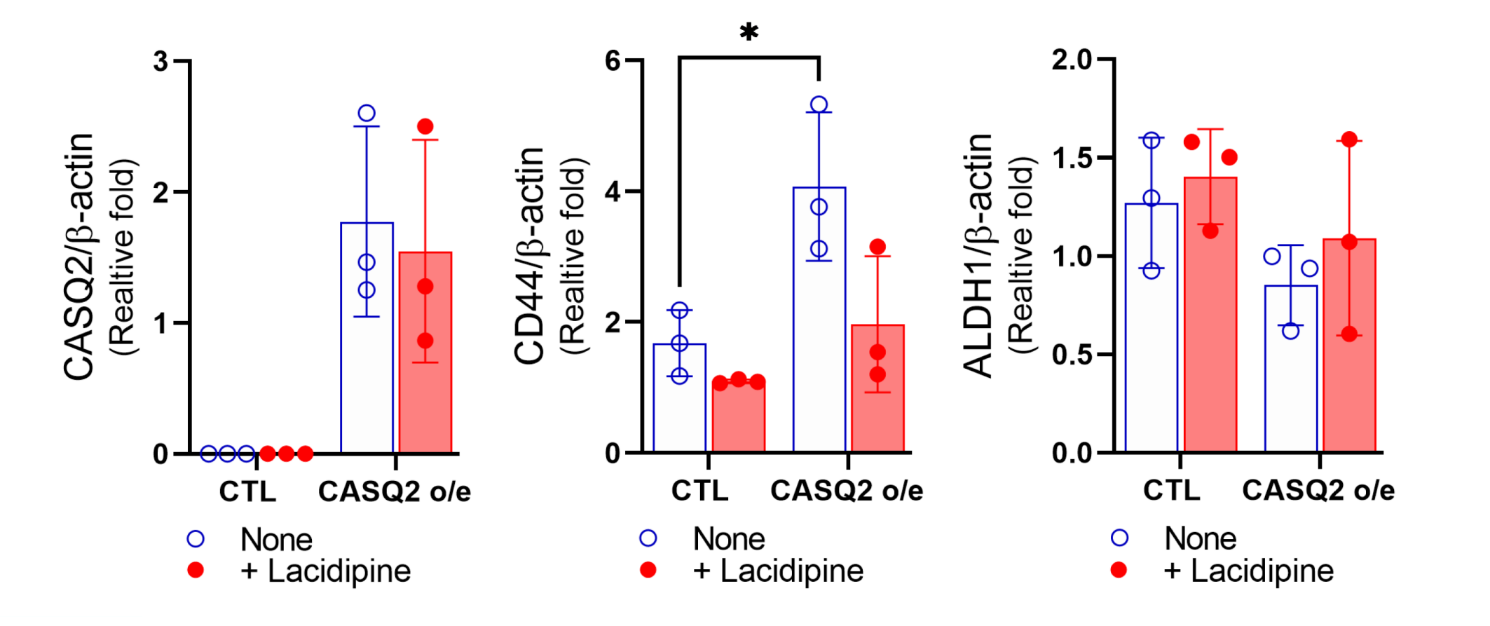


**Supplementary Figure S6. Effect of lacidipine on the expression of cancer stem cell markers in tumorspheres of breast cancer cells.** (mean ± SEM, n = 3; *^*^p* < 0.05 by Mann–Whitney *U* test)


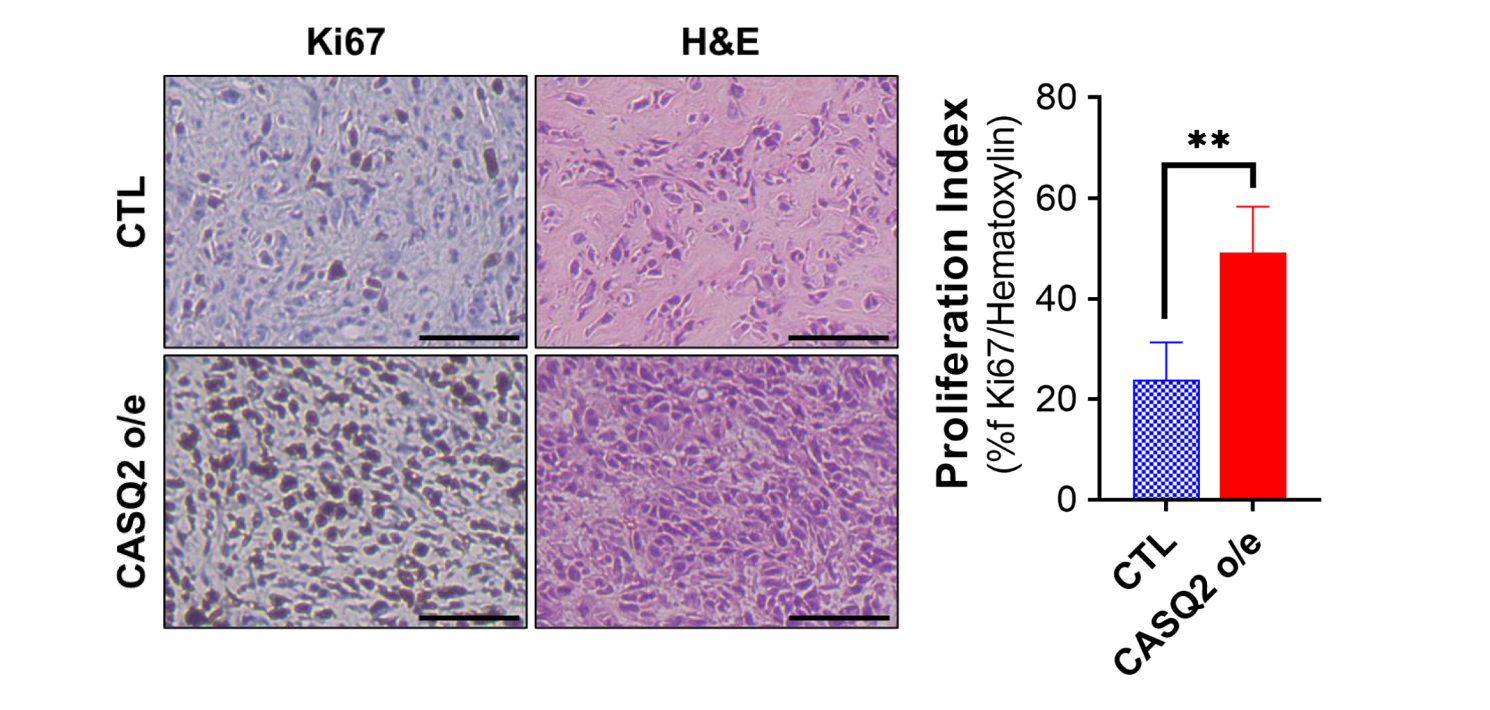


**Supplementary Figure S7. Immunohistochemical images of Ki67 in Hs578T xenograft tumor tissues.** Staining density was measured and quantified with image J (mean ± SEM, n = 5; **p* < 0.05 by the two-tailed Student’s *t*-test). Scale bar = 100 µm.


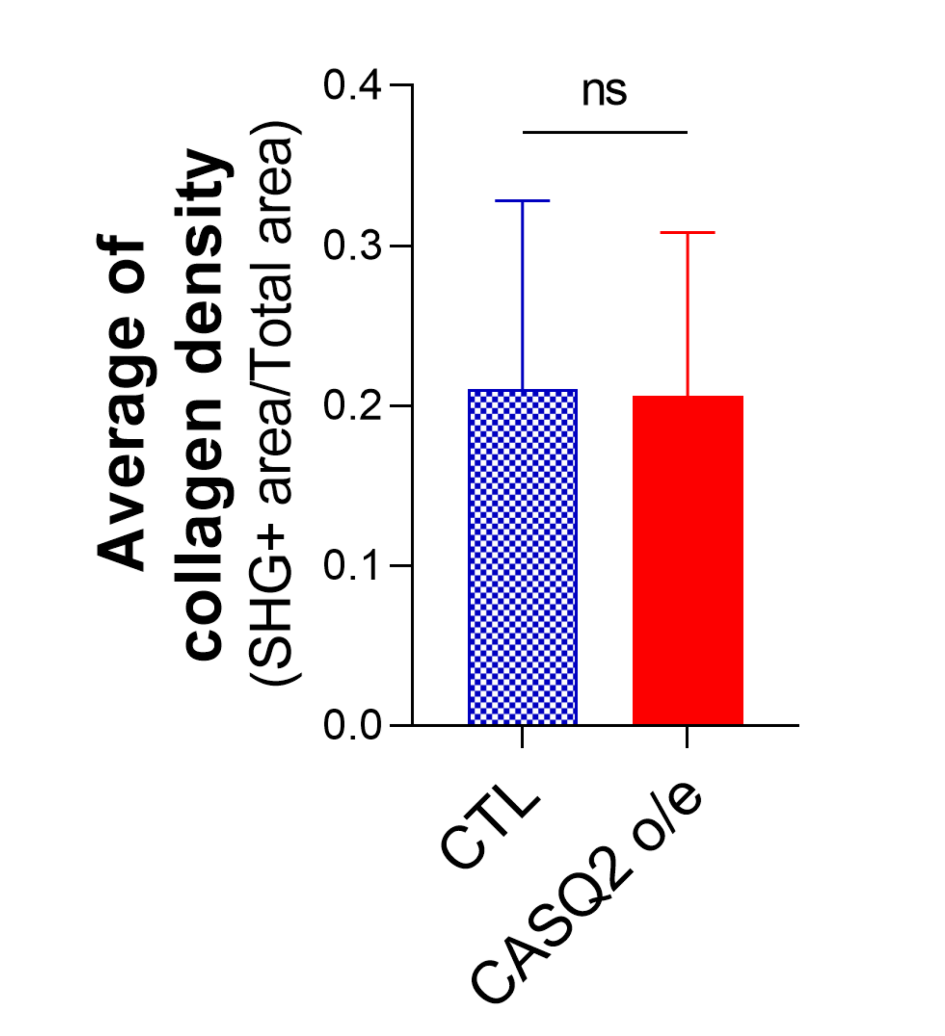


**Supplementary Figure S8. SHG+ collagen density of Hs578T-CTL and Hs578T-CASQ2 o/e tumor tissues.** (means ± SEM, n = 4; ns by two-tailed Student’s *t*-test)


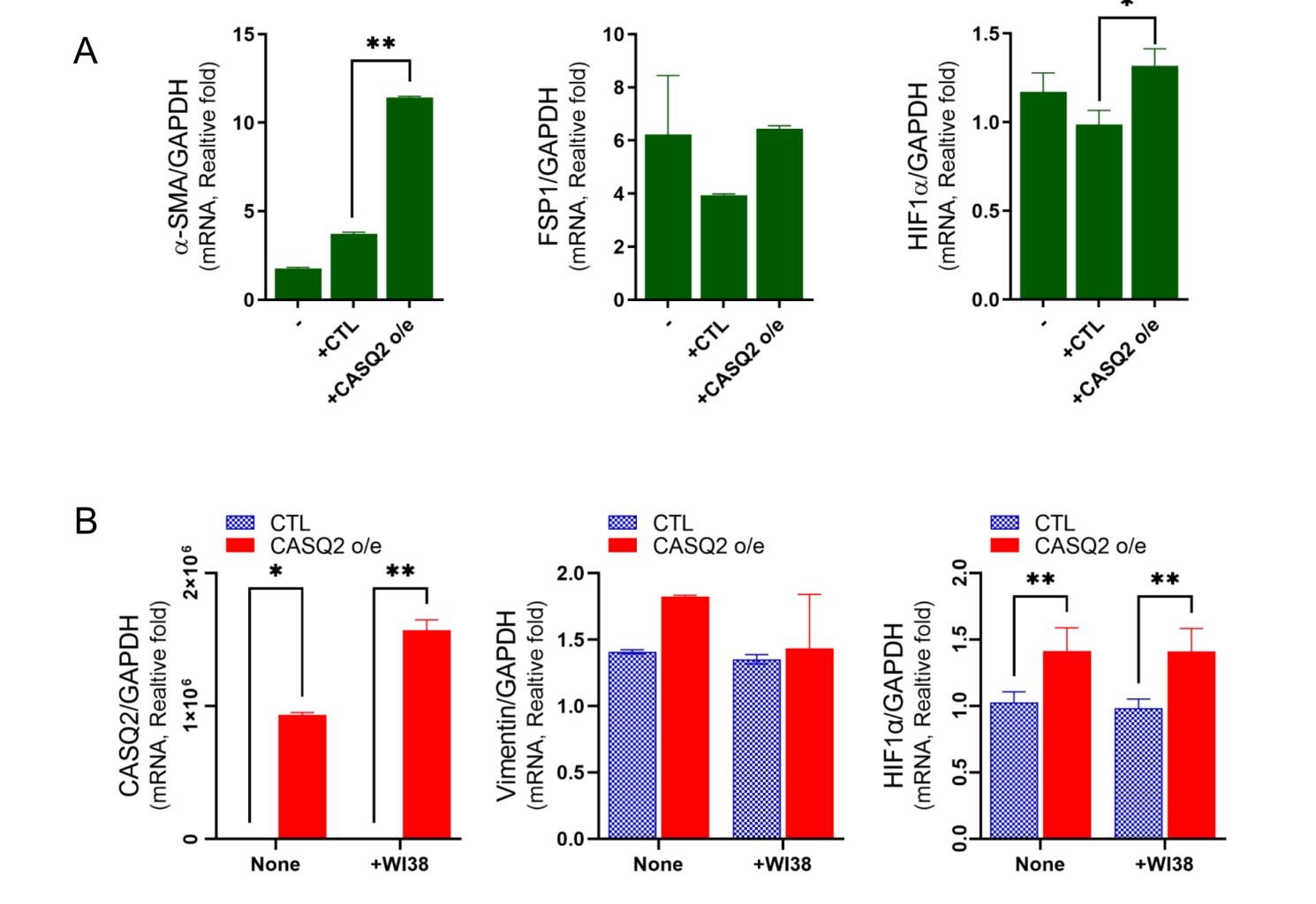


**Supplementary Figure S9. CASQ2 mediates the conversion of stromal cells to cancer-associated fibroblasts.** (A) mRNA level of αSMA, FSP, and HIF1α in WI-38 cells after indirect coculture with Hs578T-CTL or Hs578T-CASQ2 o/e cells. (B) mRNA and protein expression levels of CASQ2, Vimentin, and HIF1α in Hs578T-CTL or Hs578T-CASQ2 o/e cells indirectly cocultured with WI-38 (means ± SEM, n = 3; **p* < 0.05, ***p* < 0.01 by the multiple *t*-test).
