# Supplementary material for: Calsequestrin 2 overexpression in breast cancer increases tumorigenesis and metastasis by modulating the tumor microenvironment
Source: Mol Oncol. 2021 Nov 14;16(2):466–84. doi: 10.1002/1878-0261.13136 (PMC8763655; doi:10.1002/1878-0261.13136)
Supplement: Supplementary file 2 — Table S1. Proportions of patients with low and high CASQ2 mRNA levels according to clinicopathologic parameters of TCGA dataset. [file MOL2-16-466-s001.docx]

**Roles of calsequestrin 2 on tumor microenvironment, tumorigenesis, and metastasis of breast cancer**

**Supplementary Information**

|  | No. of  Patients (%) | CASQ2 mRNA | | P value |
| --- | --- | --- | --- | --- |
|  |  | Low (<0.0) | High (≥0.0) |  |
| All | 482(100) | 364(75.9) | 118(24.1) |  |
| Age |  |  |  | 0.430 |
| <50 | 116(24.1) | 87(23.9) | 29(24.6) |  |
| ≥50 | 258(53.5) | 203(55.8) | 55(46.6) |  |
| Stage |  |  |  | 0.123 |
| I | 42(8.7) | 33(9.1) | 9(7.6) |  |
| II | 143(29.7) | 103(28.3) | 22(33.9) |  |
| III | 136(28.2) | 113(31.0) | 13(19.5) |  |
| T stage. |  |  |  | 0.064 |
| 1 | 70(14.5) | 52(14.3) | 18(15.3) |  |
| 2 | 211(43.8) | 171(47.0) | 40(33.9) |  |
| >3 | 47(9.8) | 31(8.5) | 16(13.6) |  |
| N stage |  |  |  | 0.289 |
| N0 | 146(30.3) | 118(32.4) | 28(23.7) |  |
| N1 | 120(24.9) | 92(25.3) | 28(23.7) |  |
| >N2 | 62(12.9) | 44(12.1) | 18(15.3) |  |
| Molecular subtypes |  |  |  | <0.0001 |
| HR+/HER- (LumA) | 155(32.2) | 95(26.1) | 60(50.8) |  |
| HR+/HER+(LumB) | 87(18.0) | 78(21.4) | 9(7.6) |  |
| HR-/HER+(Her2) | 25(5.2) | 22(6.0) | 3(2.5) |  |
| HR-/HER-(Basal) | 74(15.4) | 68(18.7) | 6(5.1) |  |
| SED |  |  |  | <0.001 |
| More spheroid | 364(75.5) | 211(58.0) | 47(39.8) |  |
| Less spheroid | 118(24.5) | 153(42.0) | 71(60.2) |  |

Chi-square test: P value<0.05

**Supplementary Table S1.** **Proportions of patients with low and high CASQ2 mRNA according to clinicopathologic parameters of TCGA dataset.**
